# Blimp-1 molds the epigenetic architecture of IL-21–mediated autoimmune diseases through an autoregulatory circuit

**DOI:** 10.1172/jci.insight.151614

**Published:** 2022-06-08

**Authors:** Yu-Wen Liu, Shin-Huei Fu, Ming-Wei Chien, Chao-Yuan Hsu, Ming-Hong Lin, Jia-Ling Dong, Rita Jui-Hsien Lu, Yi-Jing Lee, Pao-Yang Chen, Chih-Hung Wang, Huey-Kang Sytwu

**Affiliations:** 1 Molecular and Cell Biology, Taiwan International Graduate Program, Academia Sinica and Graduate Institute of Life Sciences, National Defense Medical Center, Taipei, Taiwan, Republic of China.; 2 National Institute of Infectious Disease and Vaccinology, National Health Research Institutes, Miaoli, Taiwan, Republic of China.; 3 Department and Graduate Institute of Microbiology and Immunology, and; 4 Graduate Institute of Life Sciences, National Defense Medical Center, Taipei, Taiwan, Republic of China.; 5 Department of Microbiology and Immunology, School of Medicine, College of Medicine, Kaohsiung Medical University, Kaohsiung, Taiwan, Republic of China.; 6 Institute of Plant and Microbial Biology, Academia Sinica, Taipei, Taiwan, Republic of China.; 7 Department of Medicine, Washington University School of Medicine in St. Louis, St. Louis, Missouri, USA.; 8 Department of Otolaryngology-Head and Neck Surgery, Tri-Service General Hospital, National Defense Medical Center, Taipei, Taiwan, Republic of China.

**Keywords:** Autoimmunity, Cell Biology, Autoimmune diseases, Diabetes, Inflammatory bowel disease

## Abstract

Positive regulatory domain 1 (*PRDM1*) encodes B lymphocyte–induced maturation protein 1 (BLIMP1), also known as a master regulator of T cell homeostasis. We observed a negative relationship between Blimp-1 and IL-21 based on our previous data that Blimp-1 overexpression in T cells suppresses autoimmune diabetes while Blimp-1–deficient T cells contribute to colitis in NOD mice. Reanalysis of published data sets also revealed an inverse correlation between *PRDM1* and *IL21* in Crohn’s disease. Here, we illustrate that Blimp-1 repressed IL-21 by reducing chromatin accessibility and evicting an IL-21 activator, c-Maf, from the *Il21* promoter. Moreover, Blimp-1 overexpression–mediated reduction in permissive chromatin structures at the *Il21* promoter could override IL-21–accelerated autoimmune diabetogenesis in small ubiquitin-like modifier–defective c-Maf–transgenic mice. An autoregulatory feedback loop to harness IL-21 expression was unveiled by the evidence that IL-21 addition induced time-dependent Blimp-1 expression and subsequently enriched its binding to the *Il21* promoter to suppress IL-21 overproduction. Furthermore, intervention of this feedback loop by IL-21 blockade, with IL-21R.Fc administration or IL-21 receptor deletion, attenuated Blimp-1 deficiency–mediated colitis and reinforced the circuit between Blimp-1 and IL-21 in the regulation of autoimmunity. We highlight the translation of Blimp-1–based epigenetic and transcriptomic profiles applicable to a personalized medicine approach in autoimmune diseases.

## Graphical abstract

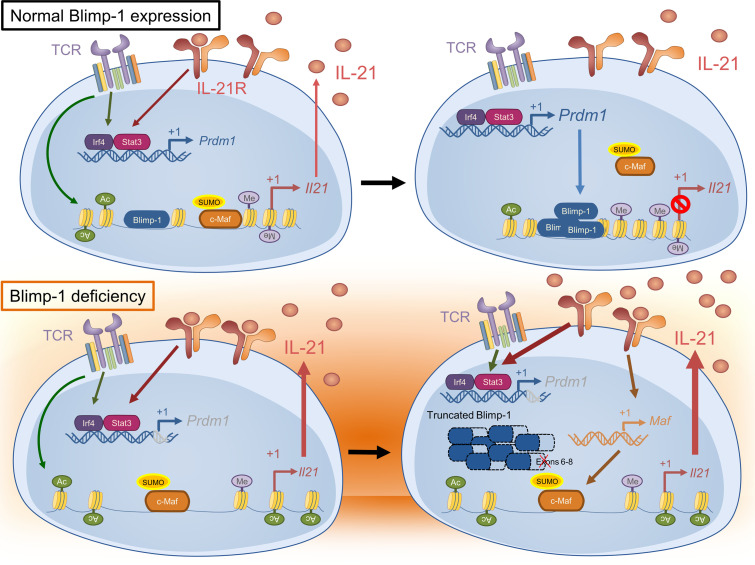

## Introduction

Genome-wide association studies have revealed numerous genetic variants conferring risk of autoimmune diseases. It has been reported that mutations in the positive regulatory domain 1 (*PRDM1*) locus, encoding B lymphocyte–induced maturation protein 1 (BLIMP1), are associated with Crohn’s disease (CD). Moreover, peripheral blood lymphocytes (PBLs) in CD patients with *PRDM1* variants are characterized by increased T cell proliferation and IFN-γ secretion. Expression of *PRDM1* in ileal biopsies from CD patients with a common CD risk-associated homozygous variant is lower than in individuals homozygous for the wild-type allele (1). In addition, 2 independent animal studies showed that lacking Blimp-1 in T cells increases the accumulation of effector/memory T cells in peripheral lymphoid organs and promotes the development of autoimmune colitis (2, 3), suggesting its suppressive role in autoimmune diseases. Furthermore, we demonstrated that T cell–specific Blimp-1 deficiency increases susceptibility to autoimmune encephalomyelitis (4) and contributes to spontaneously developed colitis (5) in NOD mice. Besides, expression of T helper 1– (Th1) and Th17-associated genes, such as *Tbx21*, *Ifng*, *Rorc*, *Il17a*, and *Il21*, is increased in T cell–specific Blimp-1 conditional knockout (CKO) NOD mice with mixed Th1/Th17 responses, resembling human CD (5). By contrast, overexpression of Blimp-1 in T cells decreases the effector functions of Th1 and Th17 cells, thereby attenuating type 1 diabetes (T1D) in these Blimp-1–transgenic (BTg) NOD mice (6). Several reports further illustrate that Blimp-1 transcriptionally represses numerous genes by multiple mechanisms (7–10). Blimp-1 binds to its consensus binding site (GAAAG) and recruits methyltransferases, deacetylases, and corepressors to form a repressor complex (7, 8). The consequent enrichment of the Blimp-1 repressor complex on target genes results in chromosome condensation by reducing active histone modification and further downregulating gene expression (7–10). Moreover, Blimp-1 has been found to repress the transcription of some activators such as nuclear factor of activated T cells 1 (NFATc1) (10) and Fos (11) or to compete with other activators such as interferon regulatory factor–1/2 (IRF-1/2) and NFATc1 for their DNA binding sites, resulting in transcriptional repression of Blimp-1–targeted genes (10, 12).

Blimp-1 expression can be induced by IL-21, a pleiotropic cytokine produced mainly by CD4^+^, CD8^+^, and natural killer (NK) T cells (13). Many immune cells such as T, B, and NK cells and dendritic cells (DCs) express IL-21 receptor (IL-21R), suggesting a broad spectrum of immunologic responses to IL-21 (14). IL-21 acts as a lymphocyte costimulator to enhance cytokine production, proliferation, differentiation, and cytotoxicity (15). IL-21 facilitates the generation of Th17 (16) and follicular helper CD4^+^ T (Tfh) cells (17). It also upregulates the expression of T-box expressed in T cells (T-bet) and IFN-γ in T cells preactivated by T cell receptor (TCR) and IL-2 stimulation (18). Overexpression of IL-21 in pancreatic β cells induces T1D in C57BL/6 mice (19) while mice deficient in IL-21 or IL-21R are resistant to T1D (19, 20). The CD4^+^ T cells of patients with T1D produce high levels of IL-21, and this increase in IL-21 production is positively associated with a higher frequency of Th17 and Tfh cells within the memory cell population (21, 22). Similarly, the populations of IL-21–, IFN-γ–, and IL-17–producing CD4^+^ T cells are increased in patients with inflammatory bowel disease (IBD), including both CD and ulcerative colitis, compared with healthy controls (23). Interestingly, IL-21 blockade in T cells from patients with IBD downregulates IL-17 and IFN-γ production (24, 25). Taken together, these data suggest that IL-21 is a potential therapeutic target in a wide range of autoimmune diseases (26, 27). However, the transcriptional regulation of IL-21 during autoimmune responses remains elusive.

It has been reported that some transcription factors, such as NFATc2 (28), c-Rel (29), and c-Maf (30), can transactivate *Il21* expression. Notably, c-Maf binds to the Maf recognition element (MARE) (30) and enhances IL-21 production in CD4^+^ T cells (31, 32). Recently, we demonstrated that small ubiquitin-like modifier (SUMO)ylation site-mutated c-Maf (KRc) preferentially transactivates *Il21* compared with *Il4* and *Il10*, by decreasing the recruitment of death-associated protein/histone deacetylase (HDAC) 2 and increasing the recruitment of the coactivators cAMP response element-binding protein–binding protein (CBP) and p300 to the MARE motif in the *Il21* promoter (32). Moreover, increased IL-21 promotes diabetogenesis in KRc-transgenic NOD mice and enhances the expression of Tfh-associated molecules, such as inducible T cell costimulator, programmed cell death 1 (PD-1), and CXCR5 (32), consistent with previous observations that memory T cells from patients with T1D express elevated levels of Tfh cell markers (21, 22). By contrast, the transcription factors T-bet (28) and IRF-4 binding protein (33) repress *Il21* expression by sequestering the activators, NFATc2 and IRF-4, respectively, from their binding sites within the *Il21* promoter.

TCR signaling together with cytokines such as IL-2, IL-4, IL-10, or IL-21 induces Blimp-1 expression (13). Of note, Blimp-1 plays a critical role in an autoregulatory loop by which IL-2 induces *Prdm1* expression and subsequently represses its own expression (11). IL-2 promotes *Prdm1* expression via activation of signal transducer and activator of transcription 5 (Stat5), and the consequent Blimp-1 feeds back directly to repress *Il2* by binding to its promoter. Moreover, Blimp-1 also binds to the promoter of *Fos*, an activator of *Il2*, to further inhibit IL-2 production, indicating the presence of an autoregulatory loop between IL-2 and Blimp-1. Based on previous data that IL-21 upregulates Blimp-1 expression through Stat3 and IRF-4 (13) and Blimp-1 is negatively associated with *Il21* expression in CD4^+^ T cells (4, 5), Blimp-1 is implicated to harness IL-21 overproduction in an autoregulatory loop. Here, we provide evidence that Blimp-1 binds directly to the *Il21* promoter and represses *Il21* expression by reducing chromatin accessibility and evicting the SUMO-defective c-Maf from its binding sites. Our data demonstrate that Blimp-1 serves as a gatekeeper in a feedback loop to counterbalance IL-21 secretion in response to the autoimmune processes of colitis and T1D.

## Results

### An inverse correlation between Blimp-1 and IL-21 expression is evident in patients with CD and T cell–specific BTg or CKO mice.

**Figure 1:**
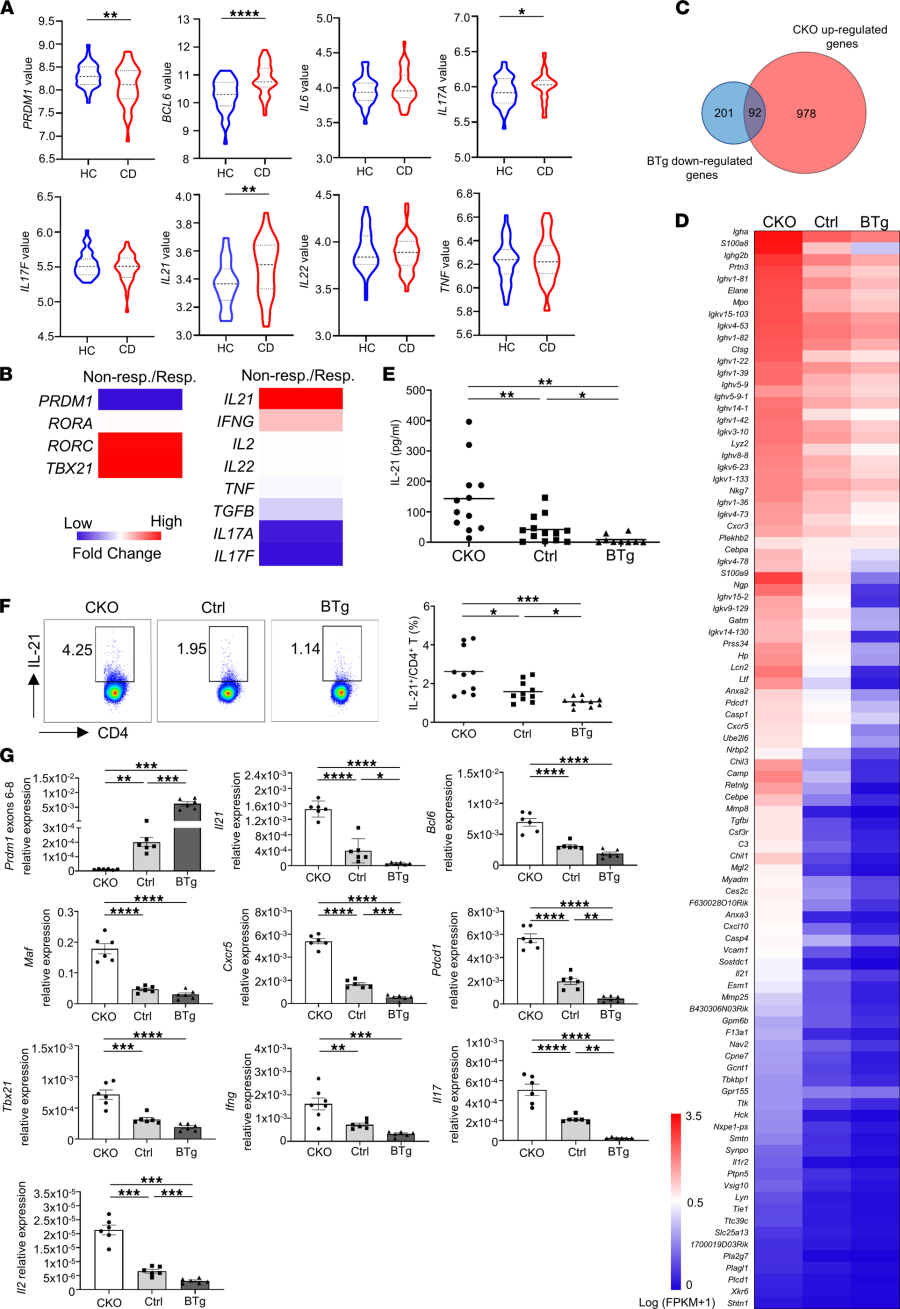
An inverse correlation between Blimp-1 and IL-21 expression is evident in patients with CD and T cell–specific BTg or CKO mice. (**A**) mRNA data set GSE126124 was downloaded from the open access GEO microarray database. The RNA expression profiling was analyzed using peripheral whole blood from 8- to 18-year-old children with Crohn’s disease (CD, *n* = 39) or healthy controls (HC, *n* = 39). The width of each curve corresponds to the approximate frequency of data points in each region. The violin plot is divided into 4 parts by 3 horizontal lines; each part contains 25% of all the data. (**B**) The analysis of GEO data set GSE171770 was used to determine RNA expression profile in sigmoid mucosal biopsy of responders (*n* = 2) and nonresponders (*n* = 2) at the end of treatment (14 weeks). (**C**–**F**) CD4^+^ T cells were isolated from 12-week-old CKO, control (ctrl), and BTg NOD mice for RNA-Seq analysis and reverse transcription quantitative PCR (RT-qPCR). (**C**) Venn diagram displaying the overlap between 2 lists of differentially expressed genes (DEGs) that were upregulated in CKO and downregulated in BTg CD4^+^ T cells compared with controls. (**D**) A heatmap representing 92 selected genes for which the fragments per kilobase of transcript per million (FPKM) were normalized by log_10_ transformation. (**E**) The level of IL-21 expression was determined in serum of CKO, control, and BTg mice using Milliplex. (**F**) Splenic IL-21–producing CD4^+^ T cells were detected in the indicated mice by flow cytometry. (**G**) The steady-state expression of the indicated genes was examined by RT-qPCR analysis. Data represent the mean ± SEM of at least 3 independent experiments; *P < 0.05; **P < 0.01; ***P < 0.001; ****P < 0.0001; significance was determined by unpaired Student’s 2-tailed *t* test (**A**) or 1-way ANOVA with Tukey’s posttest (**E**–**G**).

A previous study reported that being homozygous for the CD risk alleles in *PRDM1* locus reduces BLIMP1 expression and promotes the effector functions of PBLs, suggesting a causal relationship between expression level of BLIMP1 and CD pathogenesis (1). Considering the precise action of BLIMP1 on T cell pathogenicity remains to be fully delineated, we reanalyzed the published National Center for Biotechnology Information (NCBI) Gene Expression Omnibus (GEO) data set of peripheral blood from 8- to 18-year-old children with CD (34). We found a significant reduction in *PRDM1* expression associated with CD occurrence in contrast to upregulated *BCL6* expression, an antagonist of *PRDM1*, in patients with CD (Figure 1A). The proinflammatory cytokine profiles characterized in CD, such as *IL6*, *IL17A*, *IL17F*, *IL21*, *IL22*, and *TNF*, were further analyzed. Nevertheless, only *IL17A* and *IL21* were significantly increased in patients with CD (Figure 1A). Because Blimp-1 prevents IL-17 production in regulatory T (Treg) cells through direct regulation of the *Il17* locus (35), we hypothesize that BLIMP1 also serves as the main transcription factor negatively controlling IL-21 in T cells. The selective IL-6 trans-signaling inhibitor olamkicept leads to the reduction of a mucosal proinflammatory gene signature in patients with active IBD achieving clinical remission (36). According to aforementioned transcriptomic profiles of the clinical response to olamkicept (36), we further revealed lower *PRDM1* and higher *IL21* expression in sigmoid mucosal biopsies from nonresponders than responders in patients with CD (Figure 1B), inferring that a negative interplay between BLIMP1 and IL-21 is pivotal for patients with CD refractory to treatment.

We previously demonstrated that lacking Blimp-1 in T cells leads to the development of progressive colitis and increases susceptibility to experimental autoimmune encephalitis in NOD mice, accompanied by increased Th1 and Th17 populations (4, 5). By contrast, overexpression of Blimp-1 in T cells significantly attenuates T1D in NOD mice accompanied by reduced Th1- and Th17-related gene expression (6). To unravel how Blimp-1 systematically manipulates the global transcriptional landscape in CD4^+^ T cells to contribute to autoimmune progression, we performed RNA-sequencing (RNA-Seq) analysis in Blimp-1 CKO and BTg mice. We identified 1070 upregulated differentially expressed genes (DEGs) (log_2_ fold change > 1 and *P* value < 0.05) in CKO T cells and 293 downregulated DEGs in BTg T cells (Figure 1C and Supplemental Data 1 and 2; supplemental material available online with this article; https://doi.org/10.1172/jci.insight.151614DS1). There were 92 genes in the intersection between CKO-upregulated and BTg-downregulated DEGs (Figure 1, C and D). *Il21*, a critical hallmark of Tfh (17), was the only cytokine gene in the intersection, implying that IL-21 controls autoimmune pathogenesis in a Blimp-1–regulated manner. Moreover, we observed that the expression of *Il21*, *Cxcr5*, and *Pdcd1* negatively correlated with Blimp-1 expression (Figure 1, C and D), suggesting that an inverse relationship between the expression of Tfh cell–related genes and Blimp-1 critically modulates disease progression in CKO and BTg mice, respectively. We next evaluated the level of IL-21 protein in these mice. The serum level of IL-21 was much higher in CKO and lower in BTg mice compared with controls (Figure 1E). Our results also revealed that IL-21–producing CD4^+^ T cells were significantly increased in CKO and decreased in BTg mice compared with control mice (Figure 1F). We further characterized IL-21 production in Th0 (CD4^+^), Th1 (IFN-γ^+^CD4^+^), Th2 (IL-4^+^CD4^+^), and Th17 (IL-17^+^CD4^+^) cells from Blimp-1 CKO, control, and BTg mice. Our results revealed that the population and mean fluorescence intensity of IL-21 in Th1 and Th2 cells of CKO mice were obviously enhanced compared with control and BTg T cells (Supplemental Figure 1). Besides, although IL-21 production in Th17 cells of Blimp-1 CKO, control, and BTg mice was comparable, the frequency of IL-21–producing CD4^+^ in CKO Th17 cells was increased compared with control and BTg mice. Therefore, these results suggest that Blimp-1 can suppress IL-21 production in distinct effector T cell subsets (Supplemental Figure 1). Because it has been reported that Blimp-1 downregulates Tfh, Th1, and Th17 populations (5, 6, 37), we next determined the expression of *Il21*, *Bcl6*, *Maf*, and *Cxcr5* in the CKO and BTg CD4^+^ T cells. Expression levels of these genes were dramatically increased in CKO T cells compared with controls (Figure 1G), indicating a critical role of Blimp-1 in the inhibition of Tfh, Th1, and Th17 cell–related gene expression in NOD T cells. By contrast, *Il21*, *Cxcr5*, *Pdcd1*, *Il17*, and *Il2* transcripts were significantly decreased in BTg T cells compared with controls (Figure 1G). These data extend our previous results (5, 6) and further support the concept that Blimp-1 counterbalances IL-21 expression.

### The Blimp-1 consensus binding site is necessary and sufficient for inhibition of Il21 promoter activity through the formation of repressive chromatin structures.

**Figure 2:**
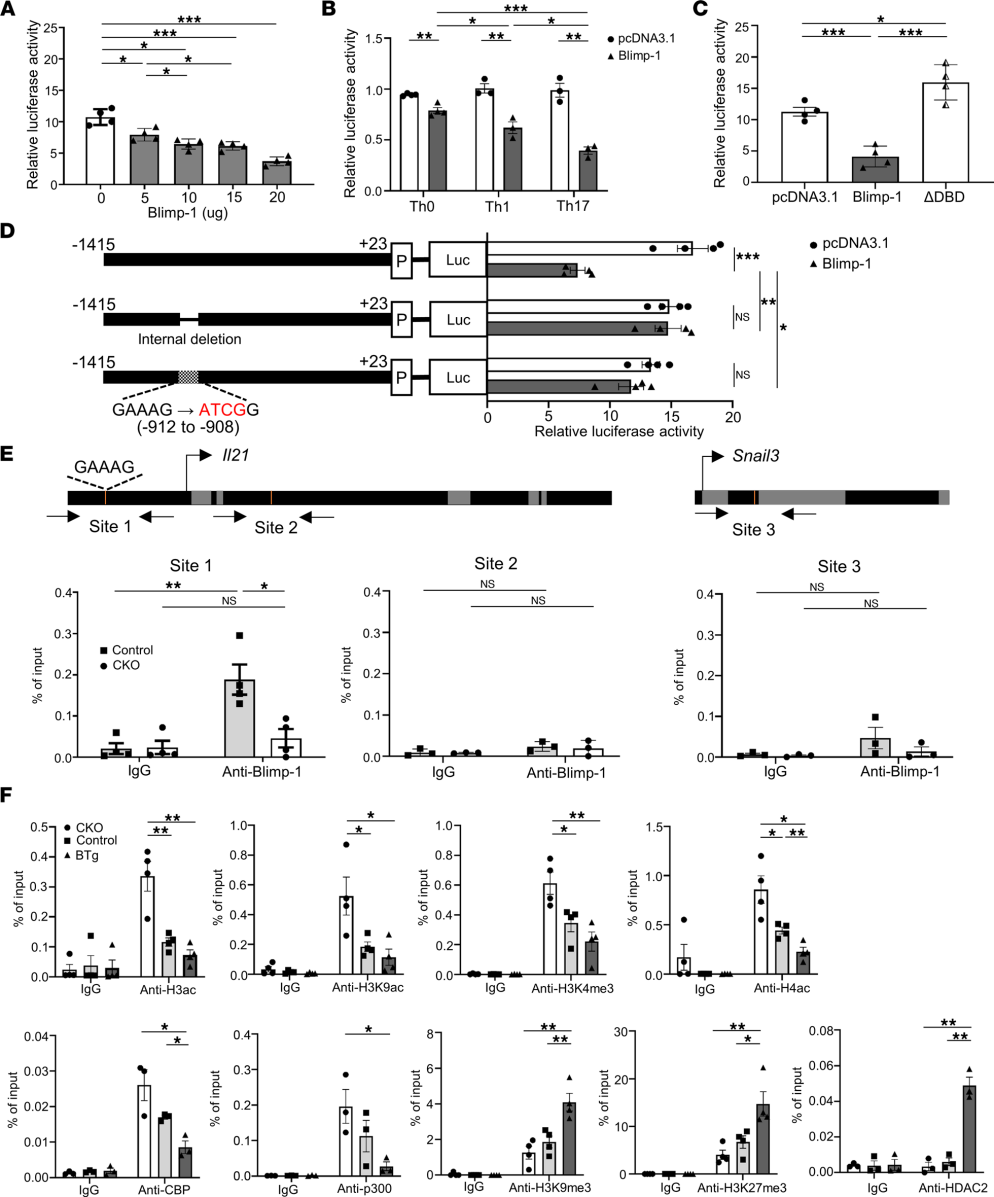
The Blimp-1 consensus binding site is essential for suppressing *Il21* promoter activity through the formation of repressive chromatin structures. (**A**) EL4 cells were cotransfected with an *Il21* promoter–driven luciferase reporter and various concentrations of Blimp-1–expressing vector as indicated. (**B**) CD4^+^CD25^–^ T cells were cultured under anti-CD3/anti-CD28 stimulation alone (Th0) or Th1- or Th17-polarized conditions. After 3 days, a luciferase assay was performed by cotransfecting the luciferase reporter and empty vectors (pcDNA3.1) or Blimp-1–expressing vectors. (**C**) Wild-type Blimp-1– or truncated Blimp-1–expressing vector (ΔDBD) was cotransfected with *Il21* promoter into EL4 cells. Then luciferase activity was determined. (**D**) Luciferase assays were performed by cotransfecting Blimp-1–expressing vectors and vectors containing an *Il21* promoter that either was full-length or contained a 5 bp (GAAAG) deletion or a site-directed mutation. (**E**) CD4^+^ T cells were isolated from indicated mice and stimulated with anti-CD3/anti-CD28 antibodies for 6 days, then restimulated with PMA and ionomycin for 4 hours, to evaluate enrichment of Blimp-1 on the *Il21* promoter with (site 1) or without (site 2) Blimp-1 binding sites and the *Snail3* locus (site 3). (**F**) CD4^+^ T cells were stimulated for 5 days and ChIP assays were performed using indicated antibodies. Data represent the mean ± SEM of at least 3 independent experiments; *P < 0.05; **P < 0.01; ***P < 0.001. Significance was determined by 1-way ANOVA (**A**–**F**).

Since Blimp-1 directly inhibits the expression of multiple genes (7–10) and we observed an inverse correlation between Blimp-1 and IL-21 expression in CKO and BTg mice (Figure 1), we proposed that Blimp-1 serves as a repressor of *Il21*. Using a bioinformatic search, we identified a potential Blimp-1 binding site in the *Il21* promoter region (NCBI reference sequence: NT_039252.1). To examine whether the binding of Blimp-1 to the *Il21* promoter represses its activity, we performed a luciferase assay by cotransfecting EL4 cells with a plasmid containing full-length *Il21* promoter (–1408 to +30) and various amounts of Blimp-1–expressing vector. Our results demonstrated that *Il21* promoter activity was suppressed by Blimp-1 in a dose-dependent manner (Figure 2A). Similarly, the promoter activities of *Il21* in Th0 or polarized Th1 and Th17 cells were also inhibited by Blimp-1 (Figure 2B), indicating that Blimp-1 transcriptionally represses IL-21 in different Th subsets. In general, the zinc finger domains of Blimp-1 are responsible for the DNA binding ability, and binding of Blimp-1 subsequently induces chromosome condensation to repress gene expression. However, there is a structurally divergent isoform of *Prdm1*, which lacks zinc domains (Blimp-1Δ7). A fraction of Blimp-1Δ7 migrates to the nucleus, colocalizes with HDAC2, and is found at sites of repressed chromatin, although it does not bind to the Blimp-1 DNA consensus site. Besides, Blimp-1Δ7 negatively regulates the *Prdm1* promoter (38). Therefore, we wonder if lack of zinc finger domains of Blimp-1 still represses gene expression. To evaluate the requirement for the DNA binding domain (DBD) of Blimp-1 for *Il21* repression, we cotransfected a DBD-truncated (deletion of exons 6–8) Blimp-1 construct and an *Il21* promoter vector into EL4 cells and measured the luciferase activity. Compared with wild-type Blimp-1, truncated Blimp-1 was not able to mediate *Il21* promoter repression (Figure 2C), supporting an essential role for the intact DBD in Blimp-1–mediated repression. Consistent with previous findings (38), truncated Blimp-1 exhibited the reverse effect, showing a higher luciferase activity than pcDNA3.1, suggesting that truncated Blimp-1 exerts an activating effect on *Il21* expression. Blimp-1 has been shown to bind to target genes that contain conserved motifs with the core consensus sequence GAAAG within their promoter and distal regions (10, 12). To investigate whether Blimp-1 downregulates *Il21* transcription through binding to these response elements, we measured the promoter activity using *Il21* luciferase reporter constructs with different modifications (Figure 2D). Our results revealed that Blimp-1 repressed approximately 60% of the promoter activity of the full-length reporter construct, whereas the activity of a promoter containing an internal deletion or a site-directed mutation of the core consensus sequence was not suppressed by Blimp-1, indicating that the presence of the consensus binding site at –912 to –908 bp is required for Blimp-1–mediated *Il21* repression (Figure 2D). We next performed chromatin immunoprecipitation (ChIP) assays to investigate whether Blimp-1 binds directly to the *Il21* promoter. Strikingly, site 1 located at –922 to –844 of the *Il21* promoter was enriched for Blimp-1 binding in control CD4^+^ T cells (Figure 2E) but not in CKO T cells in which exons 6–8 of the DBD had been deleted. By contrast, binding of Blimp-1 to site 2 at +2868 to +2938 within the *Il21* locus, a region without consensus sequence, was barely detectable, as was that at an irrelevant site 3 in the *Snail3* gene used as a negative control (Figure 2E). These data in conjunction with the inverse correlation between Blimp-1 expression and IL-21 production (Figure 1) demonstrate that *Il21* is a direct target of Blimp-1 in CD4^+^ T cells.

It has been documented that Blimp-1 excludes and/or recruits histone modifiers to its target genes, thus generating condensed chromatin for gene suppressing (7–10). In addition, *Il21* expression is associated with acetylation of histones by the histone acetyltransferases CBP and p300 (32). We therefore examined the presence of these regulatory complexes on the *Il21* promoter using ChIP assays. We observed that the *Il21* promoter in CKO T cells showed increased active histone modifications (H3ac, H3K9ac, H3K4me3, and H4ac) and histone acetyltransferases (CBP/p300) compared with control T cells, indicating an association between active histone modifications and augmented *Il21* transcription (Figure 2F). By contrast, BTg T cells showed marked reductions in all active histone modifications and acetyltransferases at the *Il21* promoter, in conjunction with significant increases in repressive modifications and deacetylases such as H3K9me3, H3K27me3, and HDAC2 (Figure 2F). In summary, our data strongly suggest that Blimp-1–mediated histone deacetylation and H3K9/H3K27 methylation at the *Il21* promoter are critical for the repression of *Il21* in CD4^+^ T cells.

### An increased accessibility of Il21 promoter is observed in CKO CD4^+^ T cells, and severity of Blimp-1 deficiency–mediated colitis can be restored by histone acetyltransferase CBP/p300 inhibitor.

**Figure 3:**
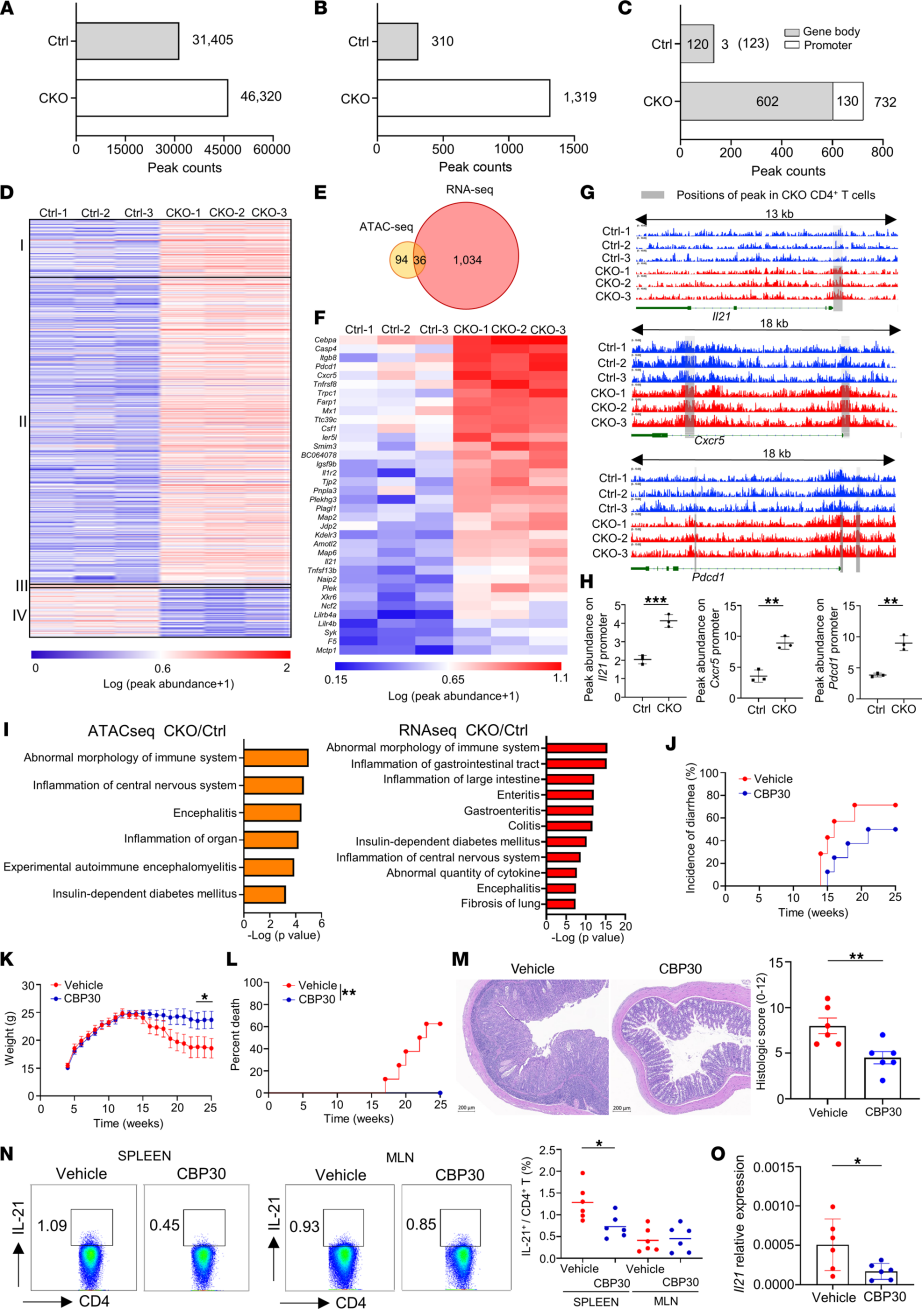
An increased accessibility of the *Il21* promoter is observed in CKO CD4^+^ T cells, and severity of Blimp-1 deficiency–mediated colitis can be restored by histone acetyltransferase CBP/p300 inhibitor. (**A**–**H**) Genome-wide ATAC-Seq data tracks for chromatin accessibility were detected for indicated T cells from 12-week-old mice. (**A**) Numbers of open chromatin regions were identified in each genotype. (**B**) DARs were identified in CD4^+^ T cells from indicated mice. (**C**) The distributions of DARs in either promoters or gene bodies from indicated cells. (**D**) Heatmap displaying DARs (peak site) in promoters (I) and gene bodies (II) in CKO CD4^+^ T cells and DARs in promoters (III) and gene bodies (IV) in control T cells. (**E**) Venn diagram illustrating the intersection of upregulated DEGs identified by RNA-Seq and the genes with DARs in promoters detected by ATAC-Seq in Blimp-1 CKO CD4^+^ T cells. (**F**) Visualization of the overlapping genes in **E**. (**G**) Representative sequencing tracks for the *Il21*, *Cxcr5*, and *Pdcd1* loci showing ATAC-Seq signals. (**H**) The peak abundance of *Il21*, *Cxcr5*, and *Pdcd1* in indicated cells. (**I**) IPA of significantly represented diseases for IL-21–dependent inflammatory disorders in CKO T cells. (**J**–**O**) CKO mice were treated with CBP30 twice a week from 12 to 25 weeks old. Incidence of diarrhea (**J**), total body weights (**K**), and survival curves (**L**) at various ages (*n* = 7 mice/group). (**M**) Representative colon sections and histological score from indicated mice at age 25 weeks. (**N** and **O**) Percentages of IL-21^+^CD4^+^ T cells (**N**) as well as *Il21* RNA expression (**O**) in indicated mice. Data represent the mean ± SEM of at least 3 independent experiments; *P < 0.05; **P < 0.01; ***P < 0.001; significance was determined using unpaired Student’s 2-tailed *t* test (**H**, **M**, and **O**), 1-way ANOVA (**N**), or log-rank test (**J**–**L**).

Consistent with the observation that Blimp-1 modulates gene suppressing through epigenetic mechanisms (8–10), we demonstrated that Blimp-1 binding to the *Il21* promoter decreases histone acetylation and increases H3K9/H3K27 methylation (Figure 2F). The status of posttranslational modifications of histones measured by ChIP assay can be used to predict gene expression. The results of an assay for transposase-accessible chromatin using sequencing (ATAC-Seq) can also determine the effects of chromatin structure modifications on gene transcription (39). To investigate insightfully whether Blimp-1 mediates *Il21* repression through downregulating the accessibility of its regulatory regions, we used ATAC-Seq to generate genome-wide chromatin accessibility maps of control and CKO CD4^+^ T cells. In total, we identified 31,405 and 46,320 high-confidence ATAC-Seq peaks representing accessible regions on chromatin in control and CKO T cells, respectively (Figure 3A). Among them, 1319 differentially accessible regions (DARs) were identified in CKO T cells and showed at least 2-fold higher ATAC-Seq abundance compared with those in control T cells (*P* value ≤ 0.05). However, only 310 DARs in control T cells showed higher signal abundance than the corresponding regions in CKO T cells (Figure 3B), indicating that Blimp-1 deficiency enhanced chromatin accessibility in T cells. We further determined the distributions of DARs in promoters (within 2 kb from the transcription start site) and gene bodies. Among these DARs, 45% of DARs (602 of 1319) in CKO T cells, similar to 38% of DARs (120 of 310) in control T cells, were located in gene bodies. However, the percentage of DARs in promoters annotated in CKO T cells (10%, 130 of 1319) was much higher than that in control T cells (1%, 3 of 310) (Figure 3, C and D, and Supplemental Data 3 and 4), suggesting that restriction of chromatin accessibility in promoter regions represents a critical mechanism by which Blimp-1 represses gene expression in CD4^+^ T cells. Our results are also supported by a previous report that increased accessibilities of promoters equate to enhancement of gene expression in memory CD8^+^ T cells (40). Because of the strong correlation between the accessibility of a promoter and the level of expression of its gene (41), we integrated ATAC-Seq (Figure 3C) and RNA-Seq (Figure 1C) data and identified 36 upregulated genes annotated from 130 DARs located in promoter regions within CKO T cells (Figure 3E), including well-known hallmarks of Tfh cells such as *Il21*, *Cxcr5*, and *Pdcd1* (Figure 3F). Moreover, both visualization of the mapped ATAC-Seq reads on *Il21*, *Cxcr5*, and *Pdcd1* (Figure 3G) and the peak abundance across these loci (Figure 3H) revealed a notable increase in the promoters in CKO T cells compared with control T cells, further supporting that the chromatin accessibility of these promoters is enhanced in the absence of Blimp-1.

We have observed a negative relationship between Blimp-1 and IL-21 in our previous reports: overexpression of Blimp-1 in T cells suppresses autoimmune diabetes (6) whereas lack of Blimp-1 in T cells results in severe colitis in NOD mice (5). To identify and/or validate whether the immune diseases and disorders potentially modulated by IL-21 are linked with Blimp-1 deficiency, we first subjected genes selected from ATAC-Seq for differential chromatin accessibility and RNA-Seq data for expression levels in CKO and control T cells to Ingenuity Pathway Analysis (IPA). Our results illustrated that these Blimp-1 deficiency–modulated and IL-21–dependent inflammatory diseases and disorders include abnormal morphology of the immune system, inflammation of the gastrointestinal tract, inflammation of the central nervous system, and insulin-dependent diabetes mellitus (Figure 3I), supporting that Blimp-1 deficiency is correlated to IL-21–based autoimmune diseases. Because the data described that the *Il21* promoter in CKO CD4^+^ T cells had increased occupancy by the histone acetyltransferase CBP/p300 (Figure 2F) that subsequently facilitated *Il21* transcription, we explored whether an interruption of CBP/p300 recruitment to the *Il21* promoter attenuated Blimp-1 deficiency–mediated colitis at the initiation of disease. We treated CKO mice with CBP30, a selective inhibitor of the CBP/p300 bromodomain (42), twice a week from 12 to 25 weeks of age, and monitored their colitogenic processes by assessing diarrhea (Figure 3J), body weight loss (Figure 3K), and mortality (Figure 3L). CKO mice receiving CBP30 exhibited milder disease and less weight loss compared with vehicle-treated controls (Figure 3, J and K). In addition, vehicle-injected mice showed high mortality rate up to 60% at 25 weeks of age whereas CBP30-treated mice were completely resistant to colitis-induced death (Figure 3L). Furthermore, histopathological assessment of colon tissues revealed less crypt damage, moderate architectural distortion, and fewer infiltrating cells in CBP30-treated mice than in controls (Figure 3M). Because of the potential immunomodulatory effects of bromodomain inhibitors on Th1, Th2, Th17, and Treg (Foxp3^+^CD4^+^) populations and subsequent autoimmune pathogenesis (42–46), we also evaluated whether CBP30-mediated protection in CKO mice is mediated by modulation of these Th subsets. Our results showed that the percentages of Th1, Th2, and Treg cells in both spleen and mesenteric lymph nodes (MLNs) were comparable between CBP30- and vehicle-injected CKO mice (Supplemental Figure 2). Although Th17 cells were not decreased in CBP30-treated mice (Supplemental Figure 2) as expected, the percentage of IL-21–producing CD4^+^ T cells in spleens (Figure 3N) and the level of *Il21* RNA transcript (Figure 3O) were significantly decreased in CBP30-treated mice compared with vehicle-treated mice. These results further support that administration of CBP30 preferentially suppresses IL-21 production in an epigenetic manner and hence ameliorates Blimp-1 deficiency–mediated colitis.

### Blocking IL-21 signaling retards colitogenic progression in Blimp-1 CKO mice.

**Figure 4:**
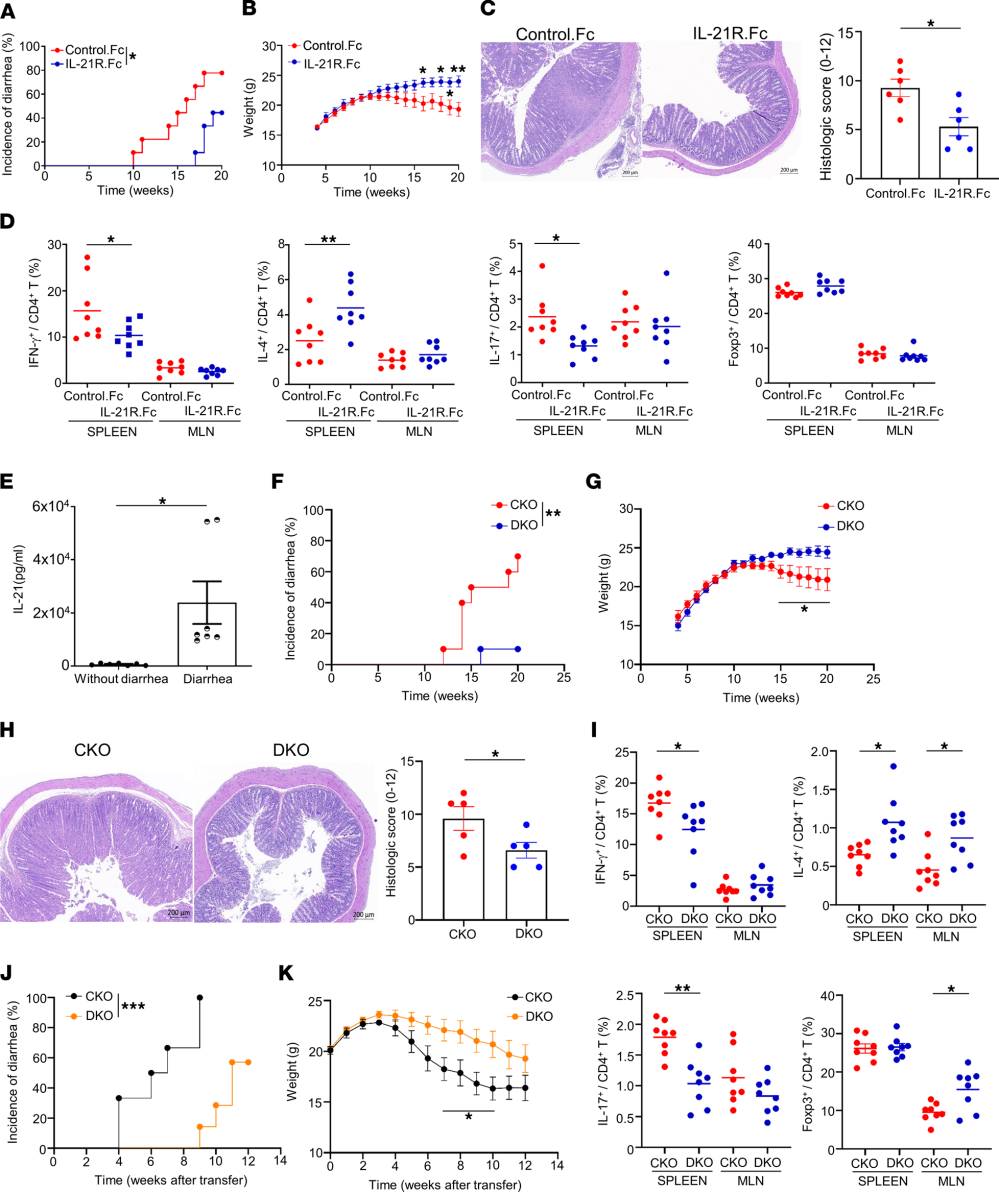
IL-21 blockade attenuates the intestinal inflammation in Blimp-1 CKO NOD mice. (**A**–**D**) CKO mice were treated with 20 mg/kg of laboratory-made recombinant IL-21R.Fc fusion protein every other day starting from 6 to 20 weeks old. Incidence of diarrhea (**A**) and total body weights (**B**) at various ages (*n* = 9 mice/group). (**C**) Representative colon sections from 20-week-old mice. (**D**) Percentages of Th1, Th2, Th17, and Treg cells in 20-week-old mice. (**E**) Serum level of IL-21 in 20-week-old CKO mice with or without diarrhea. (**F** and **G**) Colitis incidence (**F**) and total body weights (**G**) of indicated mice at various ages (*n* = 9–10 mice/group). (**H**) Representative colon sections from 20-week-old mice. (**I**) Percentages of Th1, Th2, Th17, and Treg cells in 20-week-old mice. (**J** and **K**) Diarrhea incidence (**J**) and total body weights (**K**) of NOD/SCID mice reconstituted with naive CKO or DKO CD4^+^ T cells. Data represent the mean ± SEM of at least 3 independent experiments; *P < 0.05; **P < 0.01; ***P < 0.001; significance was determined using unpaired Student’s 2-tailed *t* test (**B**–**E**, **G**–**I**, and **K**) or log-rank test (**A**, **F**, and **J**).

To investigate the critical role of IL-21 in the Blimp-1 deficiency–mediated colitogenic process and disease severity, we neutralized IL-21 in CKO mice by intraperitoneal administration of IL-21R.Fc chimeric protein. Mice receiving control.Fc still developed a wasting disease characterized by the appearance of diarrhea and a progressive loss of body weight. Strikingly, mice treated with IL-21R.Fc exhibited less severe signs of disease (Figure 4A) and no loss of body weight (Figure 4B). Histological analysis of colon tissue revealed that neutralization of IL-21 reduced lymphocyte infiltration and distortion of the crypt architecture (Figure 4C). We also evaluated the modulatory effects of IL-21 on the development of CD4^+^ T cell subsets in IL-21R.Fc–treated CKO mice. Th1 and Th17 cells were significantly decreased and Th2 cells were notably increased in the spleen, but not in MLNs, of IL-21R.Fc–treated CKO mice, whereas there was no difference in Treg cells between treated and control groups in both spleens and MLNs (Figure 4D and Supplemental Figure 3A), suggesting that IL-21 blockade–mediated alleviation of colitis is through a systemic downregulation of pathogenic Th1 and Th17 cells and a boost in the protective Th2 subpopulation. Because IL-21 has been reported to bridge the adaptive and innate immune compartments and to drive intestinal inflammation (47), we next evaluated whether IL-21 neutralization affects innate and other immune cell populations. Administration of IL-21R.Fc reduced the percentages of conventional NK (cNK) cells and macrophages but not DCs or CD8^+^ T or B cells (Supplemental Figure 4A), suggesting the involvement of a diverse range of innate and adaptive immune cells and their interactions in the pathogenesis of inflammatory colitis.

The serum level of IL-21 in CKO mice with severe colitis was significantly higher than that in age-matched diarrhea-free CKO mice (Figure 4E), indicating the critical role of IL-21 as a driving force in the colitogenic process in CKO mice. In addition to the blockade of IL-21 by IL-21R.Fc neutralization, we further explored the effects of genetic disruption of IL-21 signaling on Blimp-1 deficiency–mediated colitis by crossing IL-21R–deficient mice with Blimp-1 CKO mice to generate a double-knockout (DKO) mouse model. Around 90% of DKO mice at 20 weeks of age were resistant to diarrhea and did not exhibit weight loss whereas only 30% of CKO mice were diarrhea free at the same age (Figure 4, F and G), further validating the pathogenic role of IL-21 in Blimp-1 deficiency–induced colitis. Histological assessment also revealed less severe colonic inflammation in DKO mice compared with that in CKO mice (Figure 4H). In addition, we observed a significant decrease of Th1 and Th17 cells in spleen and a moderate decrement of Th17 population in MLNs of DKO mice, compared with that of CKO mice (Figure 4I and Supplemental Figure 3B). The percentage of Th2 cells was markedly increased in spleen and MLNs of DKO mice compared with CKO mice (Figure 4I and Supplemental Figure 3B). Besides, the population of Treg cells in MLNs was increased compared with that of CKO mice (Figure 4I and Supplemental Figure 3B), implying a positive correlation between increased MLN Treg cells and the protective phenotype in DKO mice. However, except for the B cell population, IL-21R deficiency in CKO mice did not affect other cell populations such as CD8*^+^* T cells, cNK cells, DCs, and macrophages (Supplemental Figure 4B). These results indicated that disruption of IL-21 signaling impeded colitogenesis via increasing protective Th subsets (Treg and Th2) and decreasing inflammatory Th subsets (Th1 and Th17). To further evaluate the precise impact of IL-21 signaling in CD4^+^ T cells on colitogenesis in CKO mice, we transferred naive CD4^+^ T cells from either DKO or CKO mice into NOD/SCID recipients and evaluated disease severity by monitoring stool consistency and weight loss. Mice receiving DKO naive T cells developed diarrhea starting from 9 weeks after transfer while the onset of diarrhea in mice receiving CKO T cells was observed at 4 weeks after transfer. Moreover, mice receiving DKO T cells had a lower incidence of diarrhea (Figure 4J) and less weight loss (Figure 4K) than mice receiving CKO T cells. Collectively, these results indicated that IL-21 modulates Blimp-1 deficiency–mediated colitogenic pathogenesis in a CD4^+^ T cell–autonomous manner.

### Transgenic expression of Blimp-1 represses SUMO-defective c-Maf–mediated Il21 activation and ameliorates diabetogenesis in KRcTg mice.

**Figure 5:**
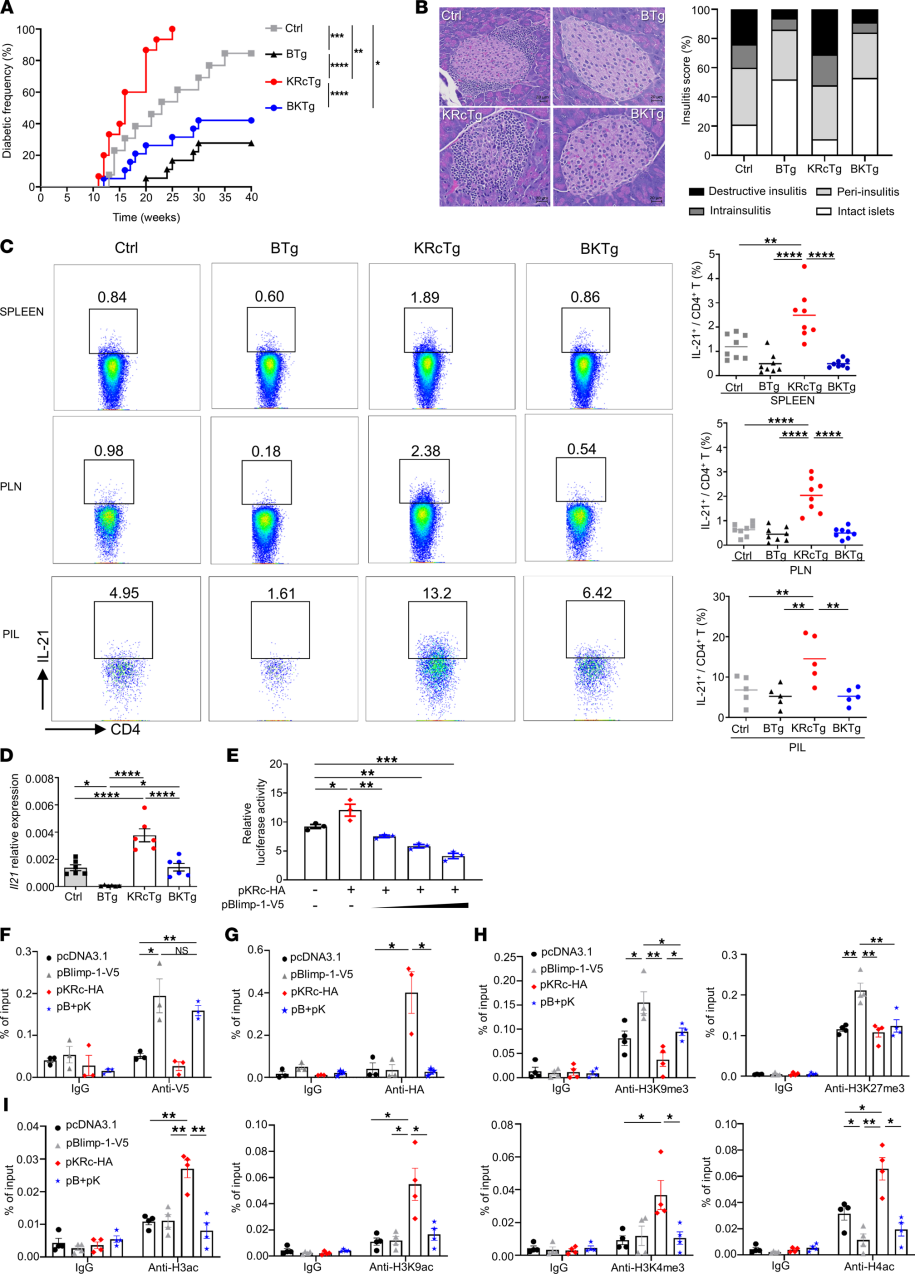
Transgenic expression of Blimp-1 represses SUMO-defective c-Maf–mediated *Il21* activation and ameliorates diabetogenesis in KRcTg mice. (**A** and **B**) Diabetes incidence (**A**) and insulitis (**B**) in 12-week-old female KRcTg, BTg, and BKTg NOD mice and their littermate controls (*n* = 13–19/group). Scale bars: 20 μm. (**C** and **D**) Frequencies of IL-21^+^CD4^+^ T cells (**C**) and *Il21* RNA expression in CD4^+^ T cells of spleens (**D**) from indicated mice. PIL, pancreas-infiltrating lymphocytes. (**E**) EL4 cells were transfected with an *Il21* luciferase reporter construct and Blimp-1-V5 alone or Blimp-1-V5 plus KRc-HA plasmids as indicated, and luciferase assays were conducted. (**F**–**I**) EL4 cells were transfected with pcDNA3.1 alone, Blimp-1-V5 alone, KRc-HA alone, or Blimp-1-V5 plus KRc-HA (pB+pK) for 24 hours. Then ChIP assays were performed using indicated antibodies. Data represent the mean ± SEM of at least 3 independent experiments; *P < 0.05; **P < 0.01; ***P < 0.001; ****P < 0.0001; significance was determined by log-rank test (**A**), 1-way ANOVA (**C**–**E**), or unpaired Student’s 2-tailed *t* test (**F**–**I**).

Although c-Maf directly transactivates IL-21 (30), which is essential for diabetogenesis in NOD mice (19, 20), our previous study demonstrated that transgenic expression of wild-type c-Maf in NOD mice enhances IL-21 production in T cells without accelerating the progression and severity of autoimmune diabetes, at least in part due to the simultaneous induction of IL-4 and IL-10. By contrast, overexpression of a SUMO-defective c-Maf transgene (KRcTg) in T cells preferentially induces excessive production of IL-21 to accelerate T1D in KRc NOD mice (32). To evaluate whether T cell-specific overexpression of Blimp-1 restrains this KRcTg-accelerated autoimmune diabetes by suppressing IL-21 production, we intercrossed BTg and KRcTg mice to generate doubly transgenic mice (BKTg) and monitored their disease progression. Consistent with our previous findings (32), we observed an accelerated onset and increased incidence of diabetes in KRcTg mice compared with control littermates (Figure 5A). Strikingly, overexpression of Blimp-1 in T cells overrode the KRcTg-augmented diabetogenesis and significantly ameliorated disease incidence in BKTg mice. Blimp-1–mediated protection was also supported by histological analyses of the pancreata of 12-week-old female NOD mice, revealing a much less severe infiltration compared with control and KRcTg mice (Figure 5B). Meanwhile, T cell–specific Blimp-1 overexpression in BKTg mice effectively downregulated the population of IL-21–producing CD4^+^ T cells in the spleen, pancreatic lymph nodes (PLNs), and pancreas-infiltrating cells (Figure 5C) and significantly limited the transcriptional expression of IL-21 (Figure 5D) compared with KRcTg mice, further supporting that the protective phenotype in BKTg mice is mainly based on Blimp-1–mediated suppression of IL-21. Other than IL-21, we also observed that the populations of Th1 cells in the spleen and PLNs were significantly increased in KRcTg mice compared with control and BTg mice. Similarly, transgenic expression of Blimp-1 downregulated IFN-γ–producing CD4^+^ T cells in BKTg mice (Supplemental Figure 5). Besides, although Th17 cells were decreased in BKTg mice compared with KRc mice, the frequency of Th17 cells in the PLNs among control, BTg, KRcTg, and BKTg mice were indistinguishable (Supplemental Figure 5). These results suggested that downregulation of IFN-γ and IL-17 may contribute to the protection in BKTg mice. Even though IL-4^+^CD4^+^ cells were decreased in spleens of BKTg mice compared with KRc mice, the incidence of autoimmune diabetes in BKTg mice was still reduced in comparison with control and KRc mice (Figure 5A), suggesting that Blimp-1 overexpression mediated a strong protection in BKTg mice and that the decrease of diabetes incidence is not directly influenced by the reduction of IL-4–producing CD4^+^ T cells. Unexpectedly, BKTg mice showed the highest population of IL-10^+^CD4^+^ T cells in the PLNs but not in spleens compared with control, BTg, and KRcTg mice (Supplemental Figure 5), implying an upregulation of IL-10 in PLNs may also contribute to the diabetic protection in BKTg mice.

Given that c-Maf effectively transactivates *Il21* expression (30, 32, 48) and Blimp-1 transcriptionally repressed IL-21 (Figure 2), we next investigated the molecular mechanism by which transgenic expression of Blimp-1 overrides KRcTg-augmented IL-21 production. We first performed a luciferase assay by cotransfecting EL4 cells with an *Il21* promoter vector and cytomegalovirus promoter–driven KRc and/or Blimp-1 expression vectors (pKRc-HA and pBlimp-1-V5, respectively). Our results demonstrated that overexpression of KRc upregulated *Il21* promoter activity, whereas the additional expression of Blimp-1 suppressed this pKRc-augmented activity in a dose-dependent manner (Figure 5E). It has been documented that Blimp-1 is a direct competitor of several gene activators, including NFATc1 and IRF-1/2 (10, 12). To characterize the binding relationship between Blimp-1 and KRc, we performed a ChIP assay after ectopic expression of these 2 genes in EL4 to determine whether the occupancy of Blimp-1 affects KRc binding to its response elements within the *Il21* promoter. Our results demonstrated significant binding of Blimp-1-V5 to its consensus binding site in the *Il21* promoter and showed that coexpressed KRc-HA did not affect the enriched level of Blimp-1-V5 on the *Il21* promoter (Figure 5F). By contrast, overexpression of Blimp-1-V5 markedly decreased KRc-HA enrichment at the MARE of the *Il21* promoter (Figure 5G). Considering that Blimp-1 overexpression did not affect the level of ectopically expressed KRc in EL4 cells (Supplemental Figure 6), these results indicated that Blimp-1 binding led to the eviction of KRc from the *Il21* promoter to downregulate its activity rather than that the loss of KRc-HA binding was caused by a Blimp-1–dependent decrease in KRc protein level. Consistent with our previous observation that BTg T cells showed significant increases in repressive modifications such as H3K9me3 and H3K27me3 (Figure 2F), ectopic expression of Blimp-1-V5 markedly enhanced the enrichment of H3K9me3 and H3K27me3 at the *Il21* promoter in EL4 cells. In addition, coexpression of Blimp-1-V5 in EL4 cells significantly augmented H3K9me3 level on the MARE of the *Il21* promoter compared with that in cells expressing KRc-HA alone (Figure 5H). By contrast, the enrichment of H3ac, H3K9ac, H3K4me3, and H4ac within the *Il21* promoter in EL4 cells cotransfected with Blimp-1-V5 and KRc-HA was much weaker than that in cells expressing KRc-HA alone (Figure 5I). These results indicated that Blimp-1 binding to the *Il21* promoter initiates installation of the repressive histone mark H3K9me3 and that removal of activating histone marks such as H3ac, H3K9ac, H3K4me3, and H4ac reduces the occupancy of MARE by KRc, thereby suppressing *Il21* promoter activity.

### IL-21 triggers Blimp-1 multiplication to suppress its own expression via an autoregulatory feedback loop.

**Figure 6:**
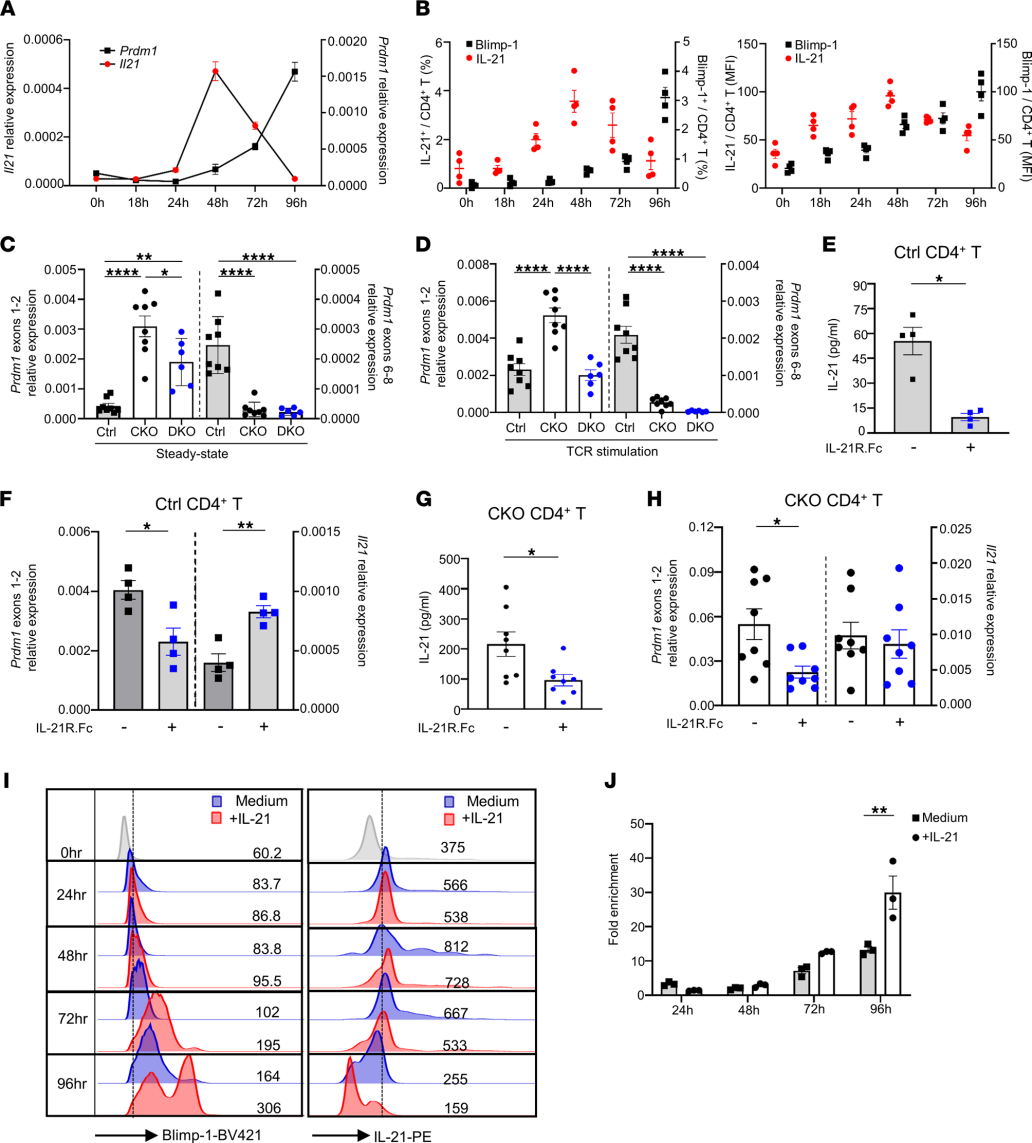
IL-21 triggers Blimp-1 multiplication to suppress its own expression via an autoregulatory feedback loop. (**A**) Expression of *Prdm1* and *Il21* RNA by control CD4^+^ T cells following TCR-mediated stimulation for the indicated times (*n* = 5). (**B**) Percentages and expression level of IL-21– and Blimp-1–producing CD4^+^ T cells at different time points. (**C** and **D**) Expression of RNA for *Prdm1* exons 1–2 and exons 6–8 in indicated T cells at steady-state (**C**) or activated (**D**) conditions. (**E** and **F**) Control CD4^+^CD25^–^ T cells were stimulated with plate-bound anti-CD3 (2 μg/mL) and soluble anti-CD28 (1 μg/mL) for 48 hours. Then, cells with original cultured medium were collected and transferred into fresh dishes containing control.Fc or IL-21R.Fc for another 48 hours. The culture medium was collected to determine IL-21 production by ELISA (**E**), and RNA expression of *Prdm1* exons 1–2 and *Il21* (**F**) were determined. (**G** and **H**) Naive CKO CD4^+^ T cells were cultured with either TCR stimulation alone or IL-21R.Fc treatment for 48 hours. The culture medium was collected to determine IL-21 production by ELISA (**G**). RT-qPCR analysis was performed to determine the expression of *Il21* and *Prdm1* exons 1–2 (**H**). (**I**) Naive control CD4^+^ T cells were stimulated with TCR alone or addition of IL-21. Blimp-1 and IL-21 expression levels were further determined by flow cytometry. (**J**) CD4^+^ T cells were stimulated for 3 days and then rested for 1 day. These preactivated T cells were treated with or without 100 ng/mL of IL-21 for the indicated times, then restimulated with PMA and ionomycin for 4 hours, and Blimp-1 enrichment on the *Il21* promoter was determined by ChIP assay. Data represent the mean ± SEM of at least 3 independent experiments; *P < 0.05; **P < 0.01; ****P < 0.0001; significance was determined using 1-way ANOVA (**C** and **D**), unpaired Student’s 2-tailed *t* test (**E**–**H** and **J**).

We demonstrated that Blimp-1 represses transcription of IL-21 by directly inducing condensation of its chromatin structure (Figure 2F and Figure 3, A–H) and by evicting c-Maf (Figure 5G). Conversely, IL-21 has been reported to be a key inducer of Blimp-1 in a Stat3-dependent and IRF-4–dependent manner (13). To further characterize the expressional kinetics and a potential regulatory loop between IL-21 and Blimp-1 in CD4^+^ T cells, we measured their RNA and protein levels at different time points. We observed a dramatic increase in *Il21* RNA from 24 hours after TCR stimulation and a 15-fold augmentation at 48 hours compared with nonactivated T cells. Subsequently, the level was markedly decreased at 72 hours and returned to baseline level at 96 hours. Notably, *Prdm1* expression gradually increased between 24 hours and 72 hours after TCR stimulation, and this increase was correlated with the decrease in *Il21* after 48 hours. The peak expression level of *Prdm1* was observed at 96 hours, corresponding to the lowest expression of *Il21* (Figure 6A). Similar to the findings of RNA expression, the peak of IL-21 protein production, in terms of both the percentage of IL-21^+^CD4^+^ T cells (Figure 6B, left panel) and their mean fluorescence intensity level (Figure 6B, right panel) appeared at 48 hours and declined gradually thereafter, whereas Blimp-1 expression increased substantially and peaked at 96 hours (Figure 6B), implying an autoregulatory loop in which a rapid induction of IL-21 after TCR stimulation (24–48 hours) mediates Blimp-1 expression and the accumulating Blimp-1 subsequently represses IL-21 production (48–96 hours). With Blimp-1 deleted, CKO T cells expressed IL-21 abundantly (Figure 1). To verify whether overproduction of IL-21 induces Blimp-1 expression, we took advantage of our CKO model in which exons 6–8 of *Prdm1*, which encode the zinc finger domains, are deleted in a Cre-dependent manner, but exons 1–2 are still expressed. Our results clearly indicated that expression of *Prdm1* exons 1–2 was dramatically increased in CKO T cells compared with control T cells (Figure 6, C and D). Noticeably, genetic disruption of IL-21 signaling in CKO CD4^+^ T cells reduced the steady-state expression of *Prdm1* exons 1–2, confirming an IL-21–mediated Blimp-1 induction (Figure 6C). Even after TCR stimulation, the expression of *Prdm1* exons 1–2 in DKO CD4^+^ T cells was also significantly lower than CKO CD4^+^ T cells (Figure 6D). Moreover, the enhanced expression of *Prdm1* exons 1–2 in CKO T cells after TCR stimulation was similar to the enhancement in expression of exons 1–2 in stimulated control T cells (Figure 6D), supporting that this indeed represents a surrogate marker for *Prdm1* expression. To verify whether an IL-21 blockade mediated Blimp-1 downregulation, control and CKO CD4^+^ T cells were treated with IL-21R.Fc, and the expression of *Prdm1* exons 1–2 was determined. The addition of IL-21R.Fc diminished the level of secreted IL-21 from control and CKO CD4^+^ T cells (Figure 6, E and G). Moreover, IL-21 blockade reduced expression of *Prdm1* exons 1–2 both in control and in CKO CD4^+^ T cells (Figure 6, F and H). However, the endogenous *Il21* expression in CKO cells was not affected by IL-21R.Fc treatment (Figure 6H). By contrast, the expression level of *Il21* in control T cells was increased, supporting that IL-21R.Fc treatment reduced *Prdm1* expression in control CD4^+^ T cells, reciprocally correlating with the *Il21* enhancement (Figure 6F). Next, to validate whether we can extrapolate those findings to the human population, freshly collected human CD4^+^ T cells from healthy blood samples were cultured under Th0 or Th1-, Th2-, Th17-, or Treg-polarized conditions for 5 days to expand these effector subsets. After 5-day culture, these polarized CD4^+^ T cells were treated with control.Fc or IL-21R.Fc for 48 hours. Our results showed that after Th0 or Th1-polarized conditions, Blimp-1–positive Th0 and Th1 cells were decreased in IL-21R.Fc–treated T cells compared with control.Fc–treated T cells, whereas the population of IL-21^+^CD4^+^ T cells was inversely increased (Supplemental Figure 7), consistent with our findings in mice (Figure 6, E and F). Moreover, IL-21 blockade apparently increased IL-21–producing cells among Th2 and Th17 cells in all human samples, and the Blimp-1–positive cells were decreased in 4 out of 5 human samples. Interestingly, under IL-21 blockade, Blimp-1–expressing Treg cells were decreased in 4 samples, but only 3 samples showed increased IL-21 production. Our data suggested that IL-21 blockade in both humans and mice resulted in a reduction of *Prdm1* expression with an inverse enhancement of *Il21* expression in distinct CD4^+^ T cell subsets. To verify whether IL-21–triggered Blimp-1 expression kinetically repressed *Il21*, we measured Blimp-1 and IL-21 expression in IL-21–stimulated T cells at different time points. Our results demonstrated that the addition of IL-21 augmented Blimp-1 expression starting from 48 hours and reaching a peak at 96 hours, correlating with a kinetic reduction in IL-21 (Figure 6I). Furthermore, TCR stimulation followed by IL-21 addition significantly increased the enrichment of Blimp-1 on the *Il21* promoter at 96 hours (Figure 6J) compared with cells without the addition of IL-21. Taken together, our results indicated that IL-21 triggers Blimp-1 to suppress its own expression via an autoregulatory feedback loop.

## Discussion

IL-21 is required for many fundamental immune processes and contributes to the development of autoimmune diseases (26, 27). Many activators such as NFATc2 (28), c-Rel (29), c-Maf (30), IRF-4 (33), BATF (49), Sp1 (50), and STAT3 (51) have been reported to regulate IL-21 gene expression, but only a small number of repressors such as T-bet (28) and Runx1 (52) have been shown to downregulate *Il21* expression. Here, we demonstrate that IL-21–augmented Blimp-1 acts in an autoregulatory feedback loop that controls IL-21 production by impairing chromatin accessibility and evicting c-Maf from the *Il21* promoter to repress IL-21 expression.

IL-2, another cytokine that develops an autoregulatory loop through Blimp-1, has been reported to promote Blimp-1 expression while Blimp-1 directly represses expression of IL-2 and its activator Fos through binding to their promoter regions (11). However, that study does not provide the detailed mechanisms by which Blimp-1 binding inhibits IL-2 and Fos expression. In our study, we also use ATAC-Seq to analyze chromatin accessibility at the *Il2* and *Fos* promoters of CKO T cells. Despite the observation that the average ATAC-Seq signal abundance of *Il2* and *Fos* promoters in CKO CD4^+^ T cells was enhanced compared with control T cells, the differences between them are less than twice (Supplemental Figure 8). Instead of the possibility that Blimp-1 changes chromatin accessibility to repress *Il2* and *Fos*, Blimp-1 could bend the DNA and/or occlude activator binding sites to inhibit their expressions. Additionally, we also observed increased chromatin accessibility at the *Pdcd1* locus in CKO T cells (Figure 3, E–H), illustrating that Blimp-1 modified chromatin accessibility to impair PD-1 expression. In parallel with the evidence that Blimp-1 is known to evict NFATc1 from its binding site to inhibit *Pdcd1* expression (10), ectopic expression of Blimp-1 restrained c-Maf from binding to the *Il21* promoter (Figure 5G). Although Blimp-1 displaces or competes with IRF1/2 for binding to the IFN-β promoter due to the similarity of their binding sequences (11), we found little similarity between the established core sequences of c-Maf and Blimp-1 binding sites on the *Il21* promoter, suggesting that competition between c-Maf and Blimp-1 for binding to the *Il21* promoter does not occur. Besides, the binding sites for Blimp-1 and c-Maf on the *Il21* promoter are at least 649 bp apart, and there is no evidence for direct interaction between c-Maf and Blimp-1, implying that Blimp-1 is unlikely to hinder c-Maf binding to the *Il21* promoter through protein-protein interaction. Instead, our results (Figure 3, C–H, and Figure 5G) indicated that Blimp-1 changed the chromatin structure and thereby evicted c-Maf from its binding site. Furthermore, the increased c-Maf in CKO T cells (Figure 1G) suggests that Blimp-1 is likely to repress *Maf* expression. Taken together, our results provide insight into the molecular mechanisms of Blimp-1–mediated IL-21 repression to control IL-21–triggered immunopathogenesis.

Although immunosuppressors such as azathioprine, TNF blockers such as etanercept, and α_4_β_7_ integrin blocker (vedolizumab) have been used to treat autoimmune diseases, the fact that full efficacy is observed in only a fraction of patients supports the necessity of genomics-based precision medicine as an approach. Indeed, we revealed that *IL21* expression is inversely correlated with *PRDM1* expression in clinical olamkicept-treated nonresponders, supporting IL-21 blockade as an alternative treatment for these nonrespondents. Given that dysregulation of IL-21 is a feature of many inflammatory autoimmune disorders, IL-21 and IL-21R are attractive therapeutic targets (26). We further treated CKO mice with IL-21R.Fc at the predisease stage (6 weeks old) and observed partial protection from colitis (Figure 4, A and B). By contrast, mice treated from the disease initiation stage (12 weeks old) were not protected from disease (Supplemental Figure 9), indicating a critical role of IL-21 early in the initiation of the autoimmune process. Although trials of several anti–IL-21 monoclonal antibodies for autoimmune diseases have been conducted (19, 53–55), the only completed phase II trial is of treatment combined with liraglutide in patients newly diagnosed with T1D (ClinicalTrials.gov NCT02443155). Clinical trials of anti–IL-21 treatment for active CD, rheumatoid arthritis, and systemic lupus erythematosus were discontinued, probably due to efficacy of IL-21 neutralization in active disease, which is consistent with our findings that therapeutic intervention for IL-21 needs to be started at the early stage of disease. A spatiotemporally controllable Blimp-1–IL-21 autoregulatory loop recapitulates the dynamics of the RNA and protein landscape in precision medicine as an emerging approach to tailor optimal therapeutic interventions for improving outcomes among autoimmune disease patients with therapy resistance.

## Methods

### Mice.

NOD/Sytwu (K^d^, D^b^, I-A^g7^, I-E^null^), B6.129/*Il21r*^–/–^, and NOD/SCID mice were initially purchased from the Jackson Laboratory. B6.129/*Il21r*^–/–^ mice were backcrossed to the NOD strain for at least 10 generations. Blimp-1 CKO, BTg, and KRcTg NOD mice were established as described previously (4, 6, 32). NOD/*Il21r*^–/–^ and Blimp-1 CKO were crossed to obtain the DKO mice. The double-transgenic mice (BKTg) were generated by crossing BTg and KRcTg mice.

### Differentiation of CD4^+^ Th cell populations.

Mouse CD4^+^CD25^–^ T cells were isolated by CD4^+^ T Cell Isolation Kit, mouse (130-104-454, Miltenyi Biotec), and stimulated with plate-coated anti-CD3 (2 μg/mL) (553058, BD Pharmingen) plus soluble anti-CD28 (1 μg/mL) (102112, eBioscience) monoclonal antibodies under Th1 (anti–IL-4, 10 μg/mL; IL-12, 10 ng/mL), Th2 (anti–IL-12, 5 μg/mL; anti–IFN-γ, 10 μg/mL; IL-4, 20 ng/mL), or Th17 (IL-6, 10 ng/mL; TGF-β, 1 ng/mL; anti–IL-4, 10 μg/mL; anti–IFN-γ, 10 μg/mL) conditions for 3 days. All cytokines were purchased from PeproTech, and anti–IL-4 (clone 11B11), anti–IL-12 (clone C17.8), and anti–IFN-γ (XMG1.2) antibodies were purchased from BioLegend. Human blood samples were provided by Yi-Wen Tsai (Department of Family Medicine, Chang Gung Memorial Hospital at Keelung, Keelung, Taiwan). Human CD4^+^ T cells were isolated from blood samples by Human CD4 T Cell Isolation Kit (130-091-155, Miltenyi Biotec), and purified cells were stimulated with Human CD3/CD28 T Cell Activator (25 μL/mL) (10971, STEMCELL Technologies) under Th0, Th1 (anti–IL-4, 10 μg/mL; IL-12, 20 ng/mL; IFN-γ, 5 ng/mL), Th2 (anti–IL-12, 10 μg/mL; anti–IFN-γ, 20 μg/mL; IL-4, 30 ng/mL; IL-2, 10 ng/mL), Th17 (anti–IL-4, 10 μg/mL; anti–IFN-γ, 20 μg/mL; IL-6, 30 ng/mL; TGF-β, 1 ng/mL; IL-1β, 10 ng/mL; IL-23, 100 ng/mL), or Treg (anti–IFN-γ, 20 μg/mL; IL-2, 10 ng/mL; TGF-β, 5 ng/mL) conditions for 5 days. All cytokines were purchased from PeproTech. Anti–IL-4 (clone MP4-25D2) and anti–IFN-γ (clone 4S.B3) antibodies were purchased from eBioscience. Anti–IL-12 (clone C17.8) was purchased from BioLegend.

### Measurement of cytokines.

Serum concentrations of IL-21 in mice were measured using a Milliplex MAP Mouse Cytokine kit (MT17MAG47PMX25BK, Merck). ELISA kits (DY594, R&D Systems) were used according to the manufacturer’s instructions for determination of IL-21 in cell culture supernatants.

### Flow cytometry.

Cells were stained with fluorochrome-conjugated antibodies for cell surface markers including human CD4 (clone OKT4, BioLegend), mouse CD4 (RM4-5/17-0042, eBioscience), CD8 (53-6.7/100712, BioLegend), B220 (clone RA3-6B2, BioLegend), I-A/I-E (M5/114.15.2, BioLegend), CD11c (clone N418, BioLegend), CD11b (clone M1.70, BioLegend), F4/80 (clone BM8, BioLegend), Lineage kit (catalog 133307, BioLegend), and NKP46 (clone 29A1.4, BioLegend). For intracellular cytokine staining, cells were stimulated with PMA (0.2 μg/mL), ionomycin (0.5 μg/mL), and monensin (2 μM) for 4 hours, then stained for surface markers. Cells were fixed, permeabilized, and further stained with fluorochrome-conjugated antibodies against human IL-21 (clone 3A3-N2, BioLegend), mouse IL-21 (clone mhalx21, Invitrogen), human IFN-γ (clone 4S.Β3, BioLegend), mouse IFN-γ (clone XMG1.2, eBioscience), human IL-4 (clone MP4-25D2, Tonbo), mouse IL-4 (11B11/504106, BioLegend), human IL-17A (clone N49-653, BD Pharmingen), mouse IL-17A (TC11-18H10.1/506915, BioLegend), or IL-10 (JES5-16E3/11-7101, eBioscience). Cells not treated with PMA, ionomycin, and monensin were used for intracellular staining for Foxp3 (FJK-16S, Invitrogen), GATA3 (TWAJ, Invitrogen), RORγt (AFKJS-9, eBioscience), Eomes (Dan11mag, Invitrogen), and Blimp-1 (5E7, BD Pharmingen) using the Foxp3/transcription factor staining buffer set (00-5523-00, eBioscience). Flow cytometry was performed on a FACSVerse (BD Pharmingen) using FlowJo software (Tree Star) for data analysis.

### RT-qPCR.

Total RNA from CD4^+^ T cells was extracted using a NucleoSpin RNA kit (Macherey-Nagel) and used for cDNA synthesis with a SuperScript III synthesis kit (Invitrogen) according to the manufacturer’s instructions. Quantitative PCR was performed using a SYBR Green method. The housekeeping gene Rps29 was used for normalization, and relative gene expression levels were calculated using the 2^–ΔΔCT^ method. The cDNA was amplified by PCR with the primer sets (Supplemental Table 1).

### Luciferase reporter assay.

EL4 cells, Th0, polarized Th1, and Th17 cells were cotransfected with an *Il21* promoter-driven luciferase reporter and expression plasmids using EL4 cell Avalanche Transfection Reagent (EZT-EL40-1, EZ Biosystems) or an Amaxa Mouse T cell Nucleofector Kit (VPA-1006, Lonza), respectively. EL4 cells are a gift from Alice Lin-Tsing Yu (Institute of Stem Cell and Translational Cancer Research, Chang Gung Memorial Hospital in Linkou, Linkou, Taiwan). A *Renilla* plasmid was also cotransfected as a control for transfection efficiency. Luciferase assays were performed 24 hours posttransfection using the Nano-Glo Dual Luciferase Reporter Assay system (N1620, Promega).

### ChIP assay.

CD4^+^ T cells were cross-linked for 10 minutes by adding formaldehyde to tissue culture medium; the final concentration of formaldehyde was 1%. Cross-linked cells were washed with phosphate-buffered saline containing protease inhibitors. Then the subsequent steps were performed using a commercial ChIP kit (MilliporeSigma). Finally, nuclear extraction was performed according to the manufacturer’s instructions. The lysates were sonicated for 20 cycles of 30 seconds each, resting on ice for 30 seconds between cycles. Cross-linked and sonicated chromatin was incubated overnight with anti–Blimp-1 (A01647, GenScript), anti–acetylated H3 (06-599, MilliporeSigma), anti–acetylated H3 (K9) (07-352, MilliporeSigma), anti–trimethyl-histone H3 (K4) (17-641, MilliporeSigma), anti–acetylated H4 (06-866, MilliporeSigma), anti-CBP (7389, Cell Signaling Technology), anti-p300 (sc-585, Santa Cruz Biotechnology), anti–trimethyl-histone H3 (K9) (ab-8898, Abcam), anti–trimethyl-histone H3 (K27) (ab-6002, Abcam), anti-HDAC2 (ab-7029, Abcam), anti-V5 (13-202, Cell Signaling Technologies), and anti-HA (A190-108A, Bethyl Laboratories) antibodies. DNA fragments were recovered using a DNA purification kit provided in the ChIP kit (MilliporeSigma) and analyzed by quantitative PCR using specific primers (Supplemental Table 2). Samples from at least 3 independent immunoprecipitations were analyzed.

### RNA-Seq and ATAC-Seq.

CD4^+^ T cells were freshly isolated from mice for the RNA-Seq or ATAC-Seq experiments. RNA-Seq experiments were performed on an Illumina HiSeq 4000, and the sequencing was outsourced to a company (TOOLS). The RNA-Seq data have been uploaded to GEO (GSE200545). ATAC-Seq was performed according to a published ATAC-Seq protocol (56). Isolated CD4^+^ T cells (5 × 10^4^) were treated with Tn5 transposase at 37°C for 30 minutes to fragment and prime DNA using the Nextera Index Kit (Illumina Nextera, FC-121-1011) according to the manufacturer’s protocol. ATAC-Seq libraries were sequenced using an Illumina HiSeq X Ten sequencer with a paired-end read.

### Bioinformatics analysis.

ATAC-Seq reads were aligned to the mouse reference genome (mm10) using Bowtie2 (57). Peak calling was performed using MACS2 (58). RNA-Seq and ATAC-Seq outcomes were uploaded to IPA software (v2019; Qiagen) for core analysis and to identify genes significantly involved in IL-21–related diseases and disorders in Blimp-1 CKO mice.

### Disease monitoring and histological examination in colitis.

Colitis was evaluated both by clinical parameters and by monitoring diarrhea and body weight. Mice were monitored twice a week from 4 weeks old for weight loss, the appearance of stool, and rectal prolapse. The mice were euthanized to remove colon tissues. The tissues were fixed in 10% formalin and stained with H&E. Colon sections were assessed individually for severity of inflammation, and histological scoring was performed in a blinded manner. Histologic damage was quantified using the histologic scoring systems described previously (5).

### Assessment of diabetes and insulitis.

The urine glucose concentration of mice was measured weekly using Chemstrips (Roche, Boehringer Mannheim). Diabetes was defined as glycosuria above 500 mg/dL on 2 consecutive tests. For histological analysis, pancreata were harvested from 12-week-old female NOD mice and fixed overnight with 10% formalin, according to the procedure previously described (6). The severity of insulitis was scored blindly on sections stained with H&E.

### Adoptive cell transfer.

Naive CD4^+^ T cells were enriched from 6- to 8-week-old CKO and control mice using a Naive CD4 T Cell Isolation Kit (130-104-453, Miltenyi Biotec). Enriched cells were counted, and 5 × 10^6^ cells were adoptively transferred into NOD/SCID recipient mice by intraperitoneal injection. The recipient mice were weighed immediately after T cell transfer and then twice a week thereafter. Their clinical signs were assessed by monitoring weight loss and change in stool consistency.

### Statistics.

Prism software (GraphPad Software) was used to perform statistical tests and to generate graphs as shown in figure legends. Data are presented as mean ± SEM of indicated *n* values. Survival analysis was performed using Kaplan-Meier with a Mantel-Cox (log-rank) test. The statistical comparisons were performed using an unpaired 2-tailed Student’s *t* test between 2 groups or 1-way ANOVA with Tukey’s posttest among multiple groups. *P* values were set as *P ≤ 0.05; **P ≤ 0.01; ***P ≤ 0.001; and ****P ≤ 0.0001.

### Study approval.

All mice were bred and maintained at the Laboratory Animal Center of the National Defense Medical Center in Taipei, Taiwan, under conventional clean conditions or in a specific pathogen–free facility accredited by the Association for Assessment and Accreditation of Laboratory Animal Care International. All experiments using live animals were reviewed and approved by the National Defense Medical Center Institutional Animal Care and Use Committee. This study and all relevant procedures were reviewed and approved by the IRB of the Chang Gung Memorial Hospital (201902205B0C502). Written informed consent was obtained from participants prior to inclusion in this study.

## Author contributions

YWL designed and conducted the experiments. YWL, SHF, and HKS wrote the manuscript. SHF, MWC, CYH, MHL, PYC, and HKS participated in experimental design and contributed to the final editing of the manuscript. JLD, RJHL, JYL, PYC, CHW, and YWL provided technical and material support.

## Supplementary Material

Supplemental data

Supplemental data set 1

Supplemental data set 2

Supplemental data set 3

Supplemental data set 4

## Version 1 05/03/2022

In-Press Preview

## Version 2 06/08/2022

Electronic publication
