# Supplementary material for: Blimp-1 molds the epigenetic architecture of IL-21–mediated autoimmune diseases through an autoregulatory circuit
Source: JCI Insight. 2022 Jun 8;7(11):e151614. doi: 10.1172/jci.insight.151614 (PMC9220827; doi:10.1172/jci.insight.151614)
Supplement: Supplemental data [file jciinsight-7-151614-s021.pdf]

Supplementary Materials for

Blimp-1 moulds the epigenetic architecture of IL-21-mediated autoimmune diseases through an autoregulatory circuit

Yu-Wen Liu[1], Shin-Huei Fu[2,3], Ming-Wei Chien[2,3], Chao-Yuan Hsu[3,4], Ming-Hong Lin[5], Jia-Ling Dong[4], Rita Jui-Hsien Lu[6, 7], Yi-Jing Lee[6], Pao-Yang Chen[6], Chih-Hung Wang[8], Huey-Kang Sytwu[1,2,3,4]

This PDF file includes:

Supplemental Figure 1

Inverse correlation of Blimp-1 and IL-21 in distinct effector $CD4^+$ T cell subsets.

Supplemental Figure 2

Th cell subsets in CBP30-treated CKO mice.

Supplemental Figure 3

IL-21 blockade decreases Th1/Th17 response and increase Th2 response.

Supplemental Figure 4

IL-21 blockade affects immune cell populations in CKO mice.

Supplemental Figure 5

Different Th subsets in KRcTg mice.

Supplemental Figure 6

Blimp-1 overexpression did not affect the level of ectopically expressed KRc in EL4 cells.

Supplemental Figure 7

IL-21 blockade in human reduces BLIMP-1 expression with an inverse enhancement of IL-21 production in distinct $CD4^+$ T cell subsets.

Supplemental Figure 8

An increased accessibility of *Il2* and *Fos* promoter are observed in CKO $CD4^+$ T cells.

Supplemental Figure 9

Blocking IL-21 signalling in late-stage of colitis cannot attenuate colitogenic progression in Blimp-1 CKO mice.

Supplemental Tables 1

Primers for RT–qPCR analysis.

Supplemental Tables 2

Primers for ChIP analysis.

Other Supplementary Materials for this manuscript include the following:

Supplemental Data 1

A list of 1070 upregulated differentially expressed genes (DEGs) in CKO $CD4^+$ T cells by RNA-seq.

Supplemental Data 2

A list of 293 downregulated DEGs in BTg $CD4^+$ T cells by RNA-seq.

Supplemental Data 3

A list of the distributions of differentially accessible regions (DARs) in promoters or gene bodies of CKO $CD4^+$ T cells by ATAC-seq.

Supplemental Data 4

A list of the distributions of DARs in promoters or gene bodies of control $CD4^+$ T cells by ATAC-seq.

Supplemental Figure

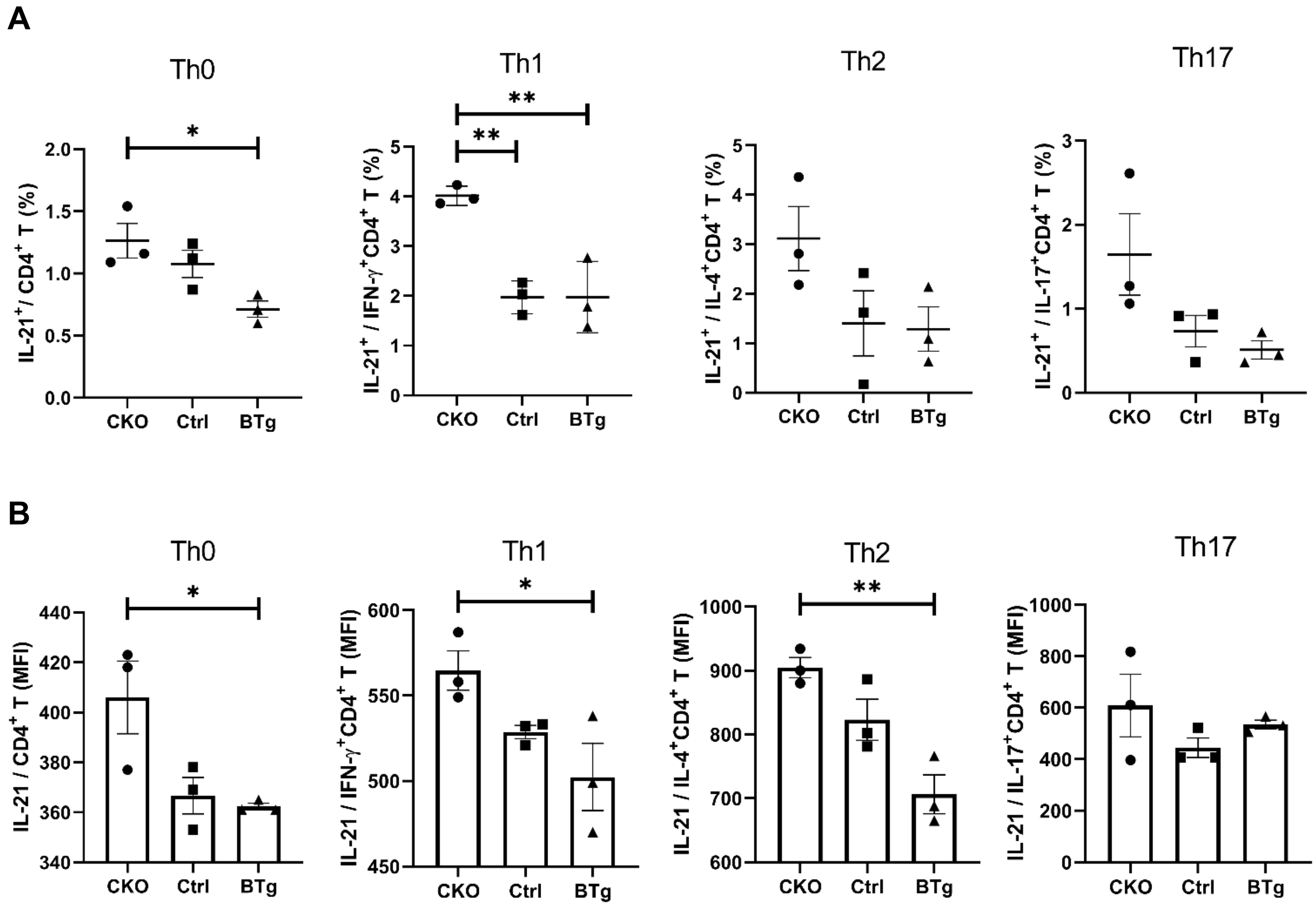

Supplemental Figure 1. Inverse correlation of Blimp-1 and IL-21 in distinct effector $CD4^+$ T cell subsets. (A-B) $CD4^+CD25^-$ T cells were isolated from indicated 12-week-old mice and cultured under Th0, Th1-, Th2-, or Th17-polarized conditions for 3 days. Summary data showing the percentages of IL-21 producing $CD4^+$ T cells (A) and mean fluorescence index (MFI) of IL-21 (B) in Th1 (IFN-γ^+CD4^+), Th2 (IL-4^+CD4^+) and Th17 (IL-17^+CD4^+) cells. Data represent the mean ± SEM of at two independent experiments; significance was determined using significance was determined by one-way ANOVA.

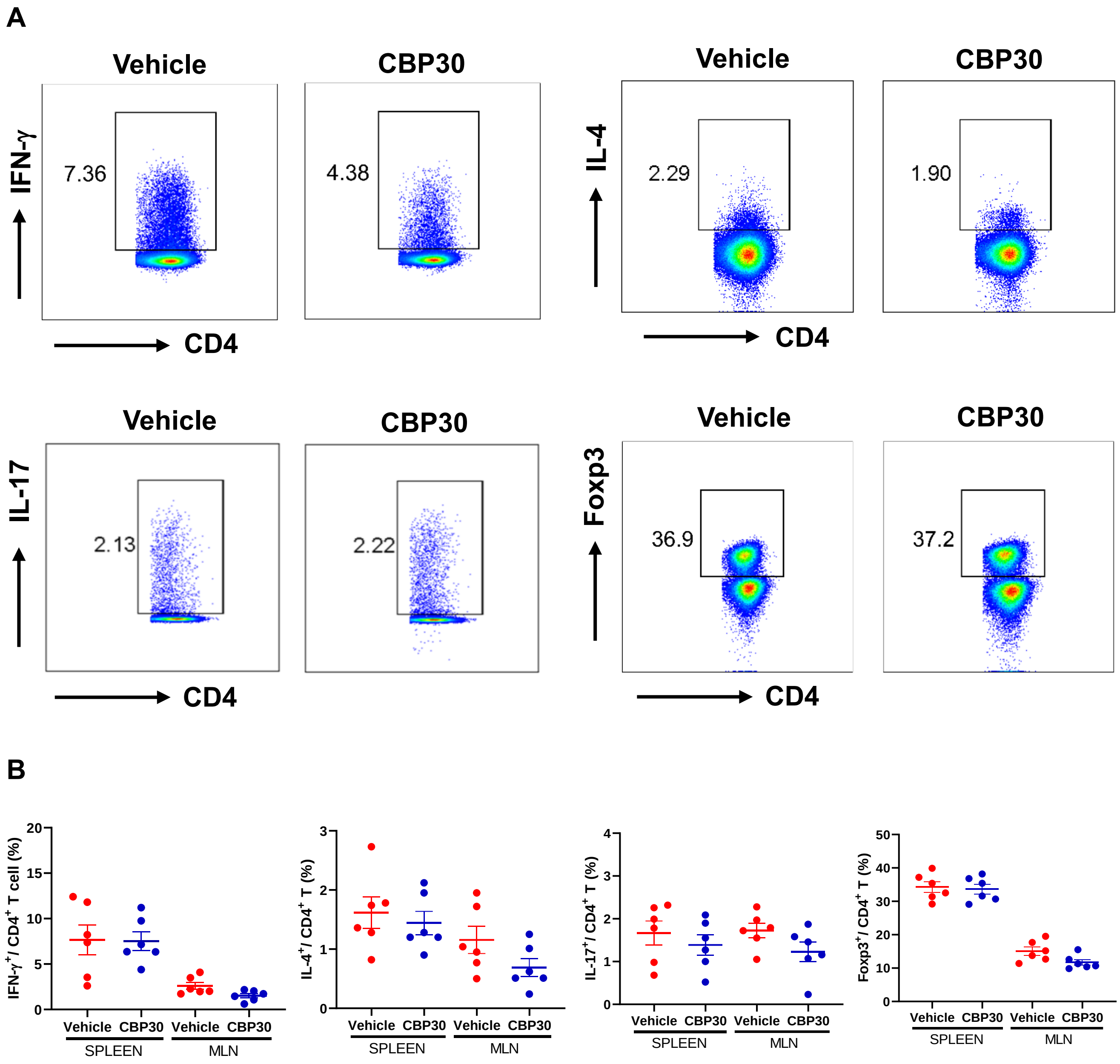

Supplemental Figure 2. Th cell subsets in CBP30-treated CKO mice. CKO mice were treated with CBP30 twice a week from 12 to 25 weeks old. (A) Representative flow cytometry dot plot graphs showing the percentage of Th1, Th2, Th17 and Treg cells in spleen from indicated mice at 25-week-old. (B) Summary data showing the percentages of indicated populations in spleen and mesenteric lymph nodes (MLN) from 25-week-old mice. Data represent the mean ± SEM of at least three independent experiments; significance was determined using significance was determined by one-way ANOVA.

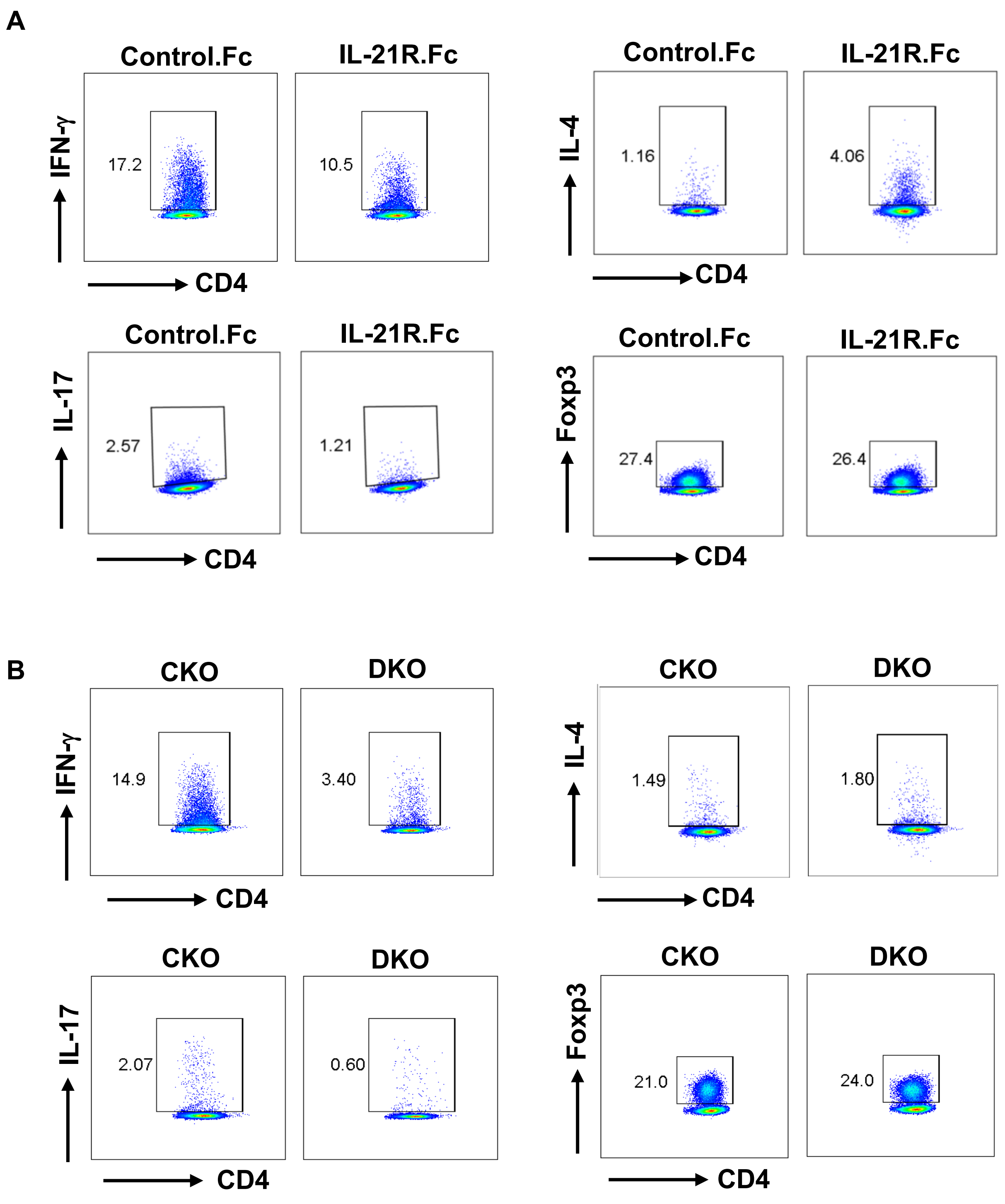

Supplemental Figure 3. IL-21 blockade decreases Th1/Th17 response and increase Th2 response. (A) CKO mice were treated with 20 mg/kg of recombinant IL-21R.Fc fusion protein every other day starting from 6 to 20 weeks old. Representative flow cytometry dot plot graphs showing the percentage of Th1, Th2, Th17 and Treg cells in spleen from indicated mice at 20-week-old. (B) Representative flow cytometry dot plot graphs showing the percentage of Th1, Th2, Th17 and Treg cells in spleen from CKO or DKO mice at 25-week-old.

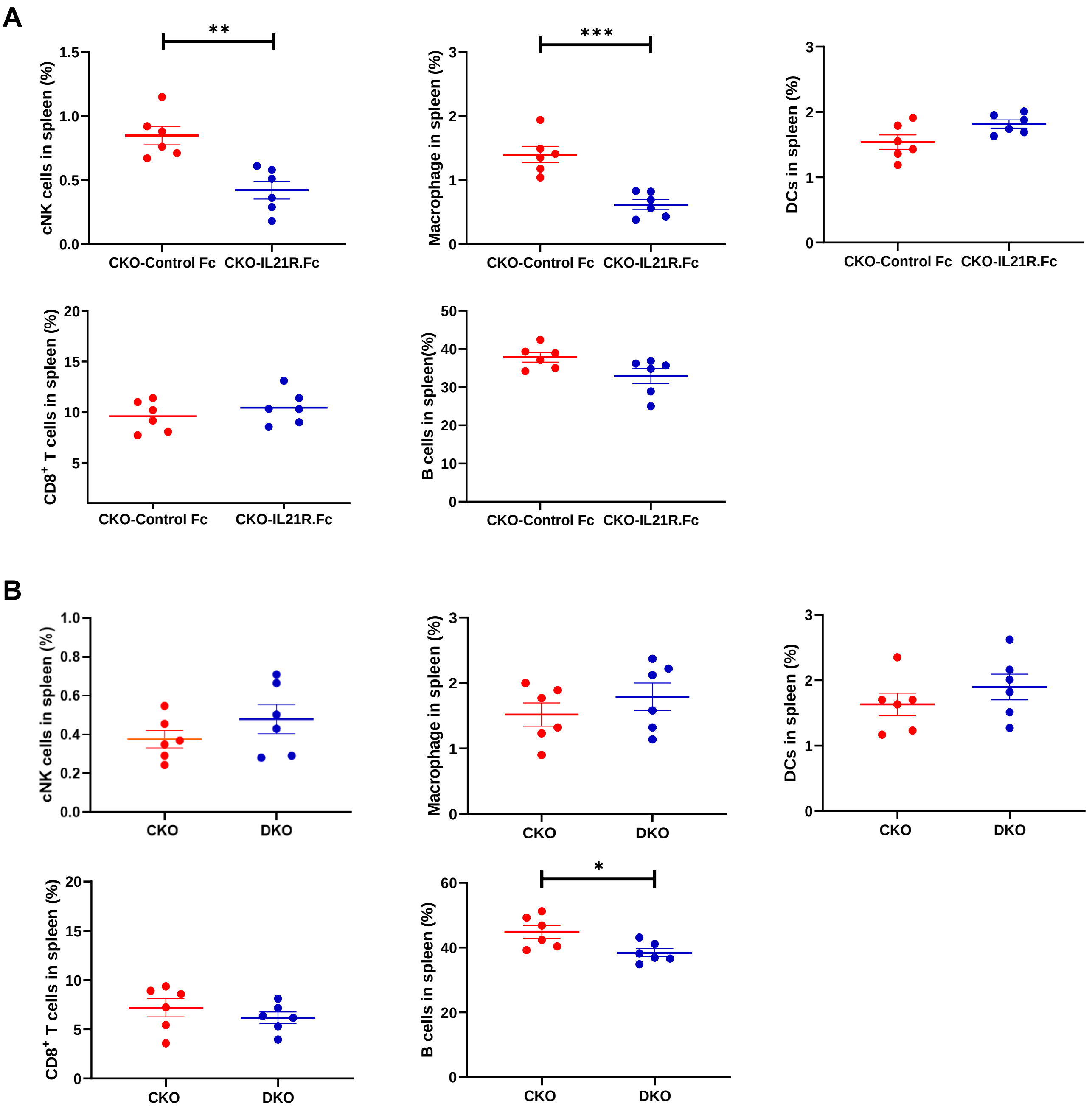

Supplemental Figure 4. IL-21 blockade affects immune cell populations in CKO mice. (A and B) Immune cell composition of spleens from 20-week-old mice was examined by flow cytometric analysis. (A) Blimp-1 CKO mice were treated with 20 mg/kg of recombinant IL21R.Fc fusion protein every other day starting from 6 weeks old to 20 weeks old. The proportion of cNK ($Lin^-GATA3^-ROR\gamma t^-NKP46^+Eomes^+$), macrophages ($CD11b^+F4/80^+$), DCs ($MHCII^+CD11c^+$), $CD8^+$ T cells and B ($B220^+$) cells in the spleens of indicated mice. (B) The percentages of cNK, macrophages, DCs, $CD8^+$ T cells and B cells in the spleens of 25-week-old CKO and DKO mice. Data represent the mean ± SEM of three independent experiments; *$p < 0.05$; significance was determined using unpaired Student's t test (A and B).

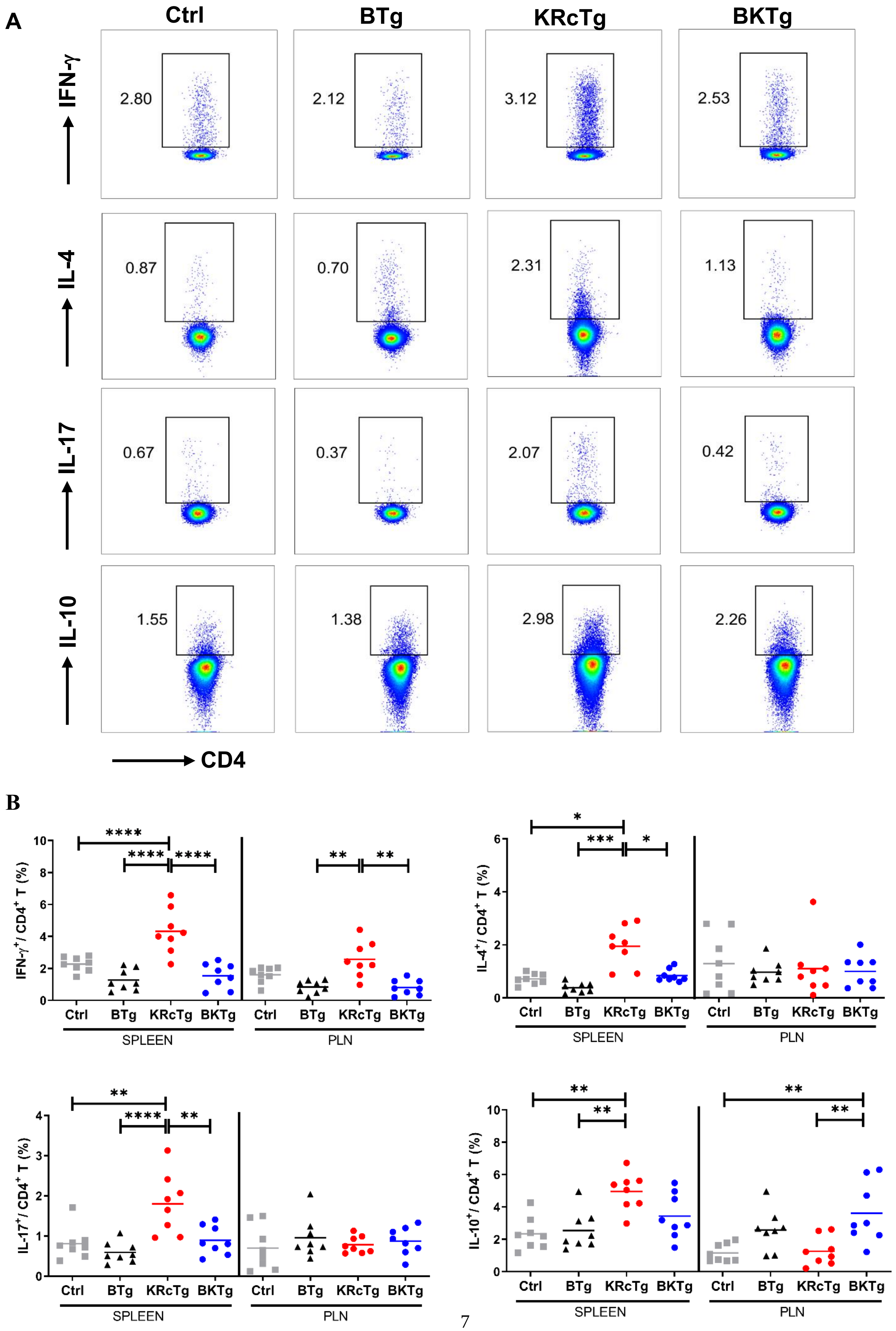

A
Ctrl
BTg
KRcTg
BKTg
IFN-γ
IL-4
IL-17
IL-10
CD4
2.80
2.12
3.12
2.53
0.87
0.70
2.31
1.13
0.67
0.37
2.07
0.42
1.55
1.38
2.98
2.26
B
IFN-γ+/ CD4+ T (%)
IL-4+/ CD4+ T (%)
IL-17+/ CD4+ T (%)
IL-10+/ CD4+ T (%)
SPLEEN
PLN

Supplemental Figure 5. Different Th subsets in KRcTg mice. (A) Representative flow cytometry dot plot graphs showing the frequencies of IFN-γ-, IL-4-, IL-17- and IL-10-producing $CD4^+$ T cells in spleen from indicated mice at 12 weeks of age. (B) Summary data showing the percentages of indicated populations in spleen and pancreatic lymph nodes (PLN) from 12-week-old mice. Data represent the mean ± SEM of at least three independent experiments; *$p < 0.05$; **$p < 0.01$; ***$p < 0.001$; ****$p < 0.0001$; significance was determined using significance was determined by one-way ANOVA.

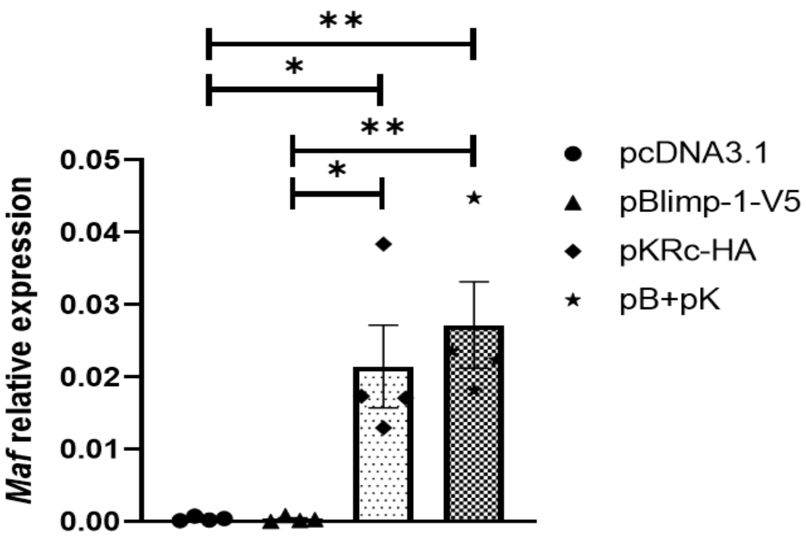

Supplemental Figure 6. Blimp-1 overexpression did not affect the level of ectopically expressed KRc in EL4 cells. EL4 cells were transfected with pcDNA3.1 alone, Blimp-1-V5 alone, KRc-HA alone or Blimp-1-V5 plus KRc-HA (pB+pK) for 24h and *Maf* RNA expression was determined by RT-qPCR. Data represent the mean ± SEM of at least three independent experiments; *$p < 0.05$; **$p < 0.01$; significance was determined by one-way ANOVA.

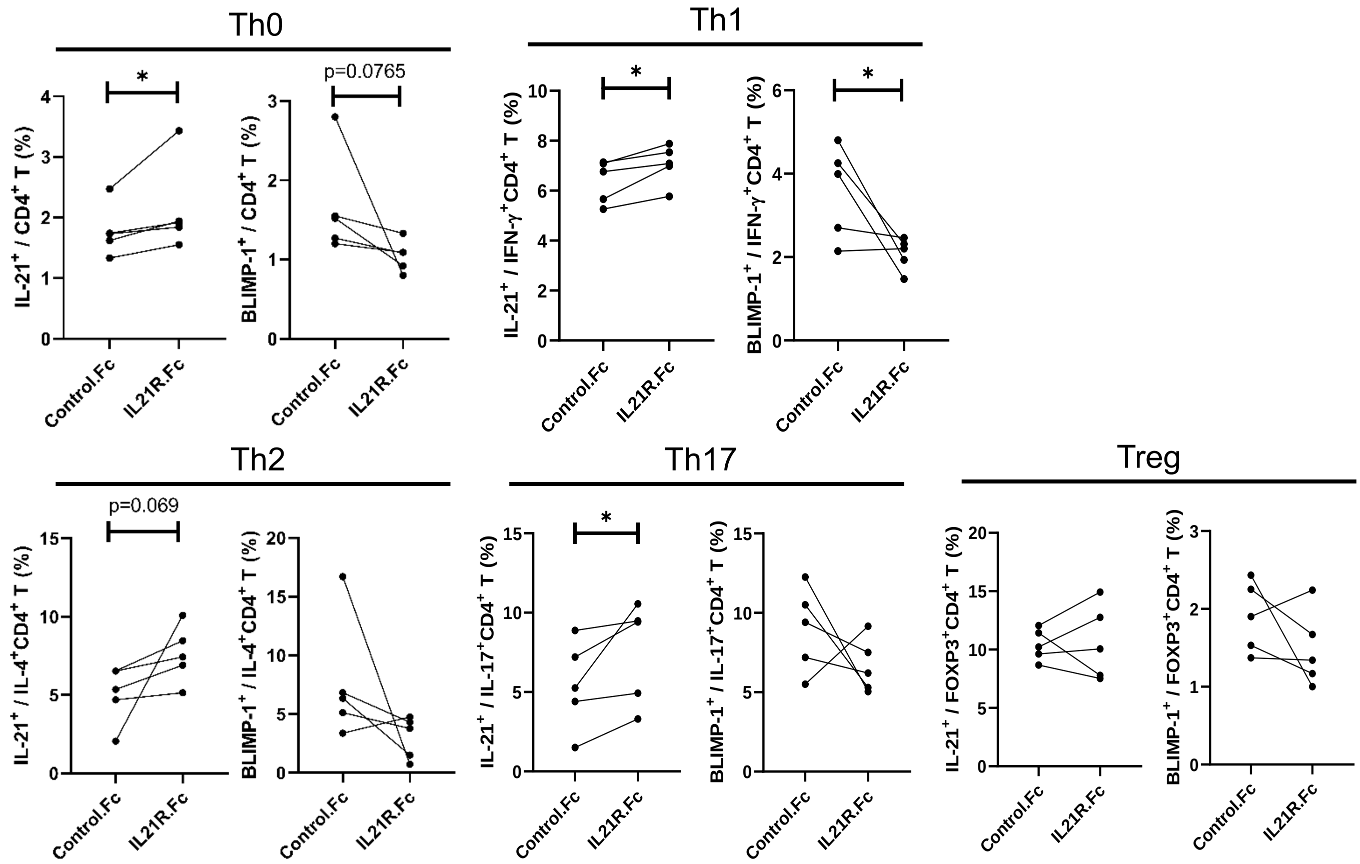

Supplemental Figure 7. IL-21 blockade in human reduces BLIMP-1 expression with an inverse enhancement of IL-21 production in distinct CD4$^+$ T cell subsets. Human CD4$^+$ T cells were isolated from healthy blood samples and cultured under Th0, Th1-, Th2-, Th17-, or Treg polarized condition for 5 days to expand these effector subsets. After 5 days culture, these polarized CD4$^+$ T cells were treated with control.Fc or IL-21R.Fc for 48 hours. Summary data showing the percentages of IL-21$^+$CD4$^+$ T cells and BLIMP-1$^+$CD4$^+$ T cells in Th0 (CD4$^+$), Th1 (IFN-γ^+CD4$^+$), Th2 (IL-4$^+$CD4$^+$), Th17 (IL-17$^+$CD4$^+$) and Treg (FOXP3$^+$CD4$^+$) cells. Data represent the mean ± SEM of at two independent experiments; *$p < 0.05$; significance was determined using paired Student’s t test.

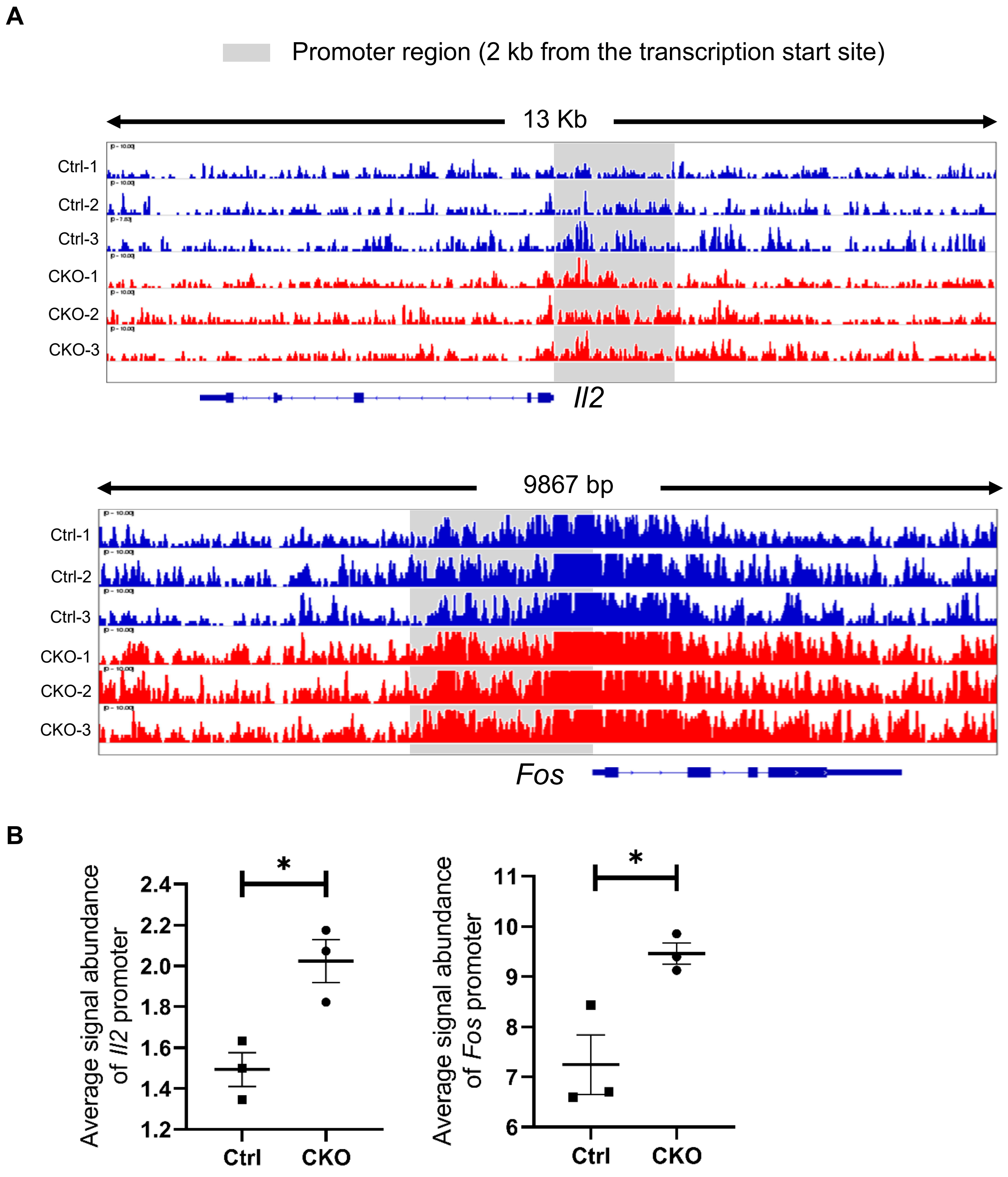

Supplemental Figure 8. An increased accessibility of *Il2* and *Fos* promoter are observed in CKO CD4$^+$ T cells. (A) Representative sequencing tracks for the *Il2* and *Fos* loci showed ATAC-seq signals. (B) The average ATAC-seq signal abundance of *Il2* and *Fos* promoters in indicated cells. Data represent the mean ± SEM of at least three independent experiments; *$p < 0.05$; significance was determined using unpaired Student's t test (B).

A

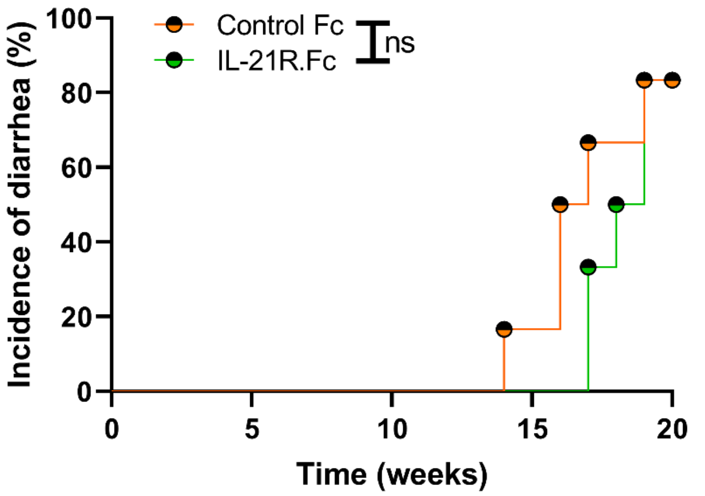

B

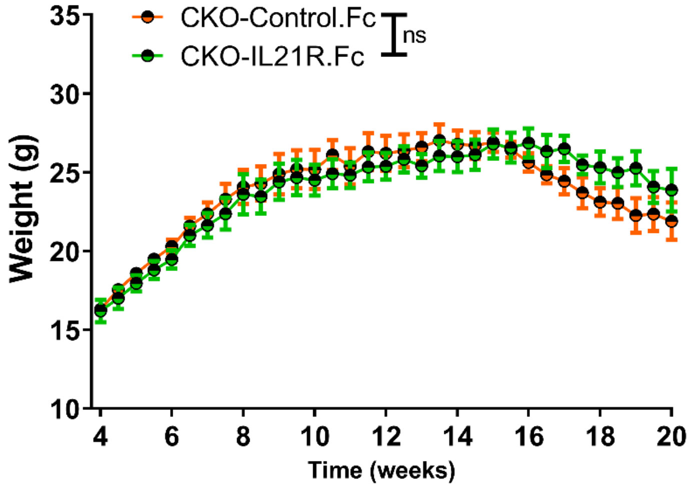

Supplemental Figure 9. Blocking IL-21 signalling in late-stage of colitis cannot attenuate colitogenic progression in Blimp-1 CKO mice. (A-B) CKO mice were treated with 20 mg/kg of recombinant IL-21R.Fc fusion protein every other day starting from 12 to 20 weeks old. Incidence of diarrhea (A) and total body weights (B) at various ages (n=9 mice/group). Data represent the mean ± SEM of three independent experiments; ns denotes no significance. significance was determined using log-rank test (A) or unpaired Student's t test (B).

Supplemental Table 1.

Primers for RT–qPCR analysis

Target genes		Sequence (5' to 3')
Prdm1 exons 6-8	F	CTCGCCCACCTGCAGAAACACT
	R	GCAGCTTCAGGTGCACAAATTGCG
Il21	F	GCTCCACAAGATGTAAAGGGGC
	R	CCACGAGGTGATGATGAATGTC
Maf	F	AAATA CGAGA AGCTG GTGAG CAA
	R	CGGGA GAGGA AGGGT TGTC
	F	TGGCCTTCTACAGTAACAGCA
	R	GCATGAATACCGCCTTAAAGGAC
Pdcd1	F	CAGCTTGTCCAACTGGTCG
	R	GCTCAAACCATTACAGAAGGCG
Bcl6	F	CTGCAGATGGAGCATGTTGT
	R	CACCCGGGAGTATTTCTCAG
Tbx21	F	GGTGTCTGGGAAGCTGAGAG
	R	CCACATCCACAAACATCCTG
Ifng	F	ATCTGGAGGAACTGGCAAAA
	R	TTCAAGACTTCAAAGAGTCTGAGG
Il17	F	TTTAACTCCCTTGGCGCAAAA
	R	CTTTCCCTCCGCATTGACAC
Il2	F	AGCAGCTGTTGATGGACCTA
	R	CGCAGAGGTCCAAGTTCAT
Prdm1 exons 1-2	F	CTTCTCTTGGAAAAACGTGTGGG
	R	TCATATCAGCGTCCTCCATGT
Rps29	F	ACGGTCTGATCCGCAAATAC
	R	AGCATGATCGGTTCCACTTG

Supplemental Table 2.

Primers for ChIP analysis

Promoter region		Sequence (5' to 3')	
Site 1 (*Il21* promoter)	F	AACTTGGTCAGAAAGGAGCATTTAC	With GAAAG sequence
	R	TGTCCGATTTATTTGTTGCTTGTATT	
Site 2 (*Il21* promoter)	F	CGACCCCTCCCCAAGCT	Without GAAAG sequence
	R	TTGCTGAGTTAACGTTTTCATTTGT	
Site 3 (*Snail* promoter)	F	ATTGCCGTCCCAGAGAAGGAT	Without GAAAG sequence
	R	TACACAGATATGGCCATTTGCC	
MARE (*Il21* promoter)	F	TGGTGAATGCTGAAAACTGGAA	Without GAAAG sequence
	R	CCCATCTGCATCTTAGAC	

Supplemental Data 1. (separate file)
A list of 1070 upregulated differentially expressed genes (DEGs) in CKO $CD4^+$ T cells by RNA-seq.

Supplemental Data 2. (separate file)
A list of 293 downregulated DEGs in BTg $CD4^+$ T cells by RNA-seq.

Supplemental Data 3. (separate file)
A list of the distributions of differentially accessible regions (DARs) in promoters or gene bodies of CKO $CD4^+$ T cells by ATAC-seq.

Supplemental Data 4. (separate file)
A list of the distributions of DARs in promoters or gene bodies of control $CD4^+$ T cells by ATAC-seq.
